# Unraveling the Synergistic Activity‐Stability Enhancement of a High‐Entropy Perovskite Air Electrode for Dual Ceramic Electrochemical Cells

**DOI:** 10.1002/advs.75100

**Published:** 2026-03-31

**Authors:** Ying Zhang, Yibei Wang, Zhilin Liu, Kuo Hu, Yaowen Wang, Zhen Wang, Xiyang Wang, Bingbing Niu, Wenquan Wang, Tianmin He

**Affiliations:** ^1^ Key Laboratory of Physics and Technology for Advanced Batteries Ministry of Education College of Physics Jilin University Changchun China; ^2^ School of Science University of Science and Technology Liaoning Anshan China; ^3^ School of Chemistry and Chemical Engineering Frontiers Science Center for Transformative Molecules Shanghai Jiao Tong University Shanghai China; ^4^ State Key Laboratory of High Pressure and Superhard Materials Synergetic Extreme Condition User Facility College of Physics Jilin University Changchun China; ^5^ State Key Laboratory of Inorganic Synthesis and Preparative Chemistry College of Chemistry Jilin University Changchun China; ^6^ Department of Applied Physics The Hong Kong Polytechnic University Hong Kong China

**Keywords:** air electrode, high‐entropy perovskite, H_2_O and CO_2_ tolerance, lattice distortion, low thermal expansion coefficient, reversible protonic ceramic cells, solid oxide fuel cells

## Abstract

Regulating the activity and stability of high‐entropy perovskite air electrodes is essential for their application in ceramic electrochemical cells, yet the underlying mechanisms remain unclear. In this work, a novel high‐entropy perovskite, Gd_0.2_Pr_0.2_Ba_0.2_Sr_0.2_Ca_0.2_FeO_3−δ_ (GPBSCF), is developed as a highly active and stable air electrode for both oxygen‐ion conducting solid oxide fuel cells (O‐SOFCs) and reversible protonic ceramic cells (R‐PCCs). It is demonstrated that high‐entropy doping increases Fe^4+^ content and structural symmetry, thereby elevating oxygen vacancy/hole concentration and enhancing catalytic activity. Concurrently, the induced lattice distortion improves structural stability and inhibits Ba/Sr surface segregation. Furthermore, the increased Fe^4+^ content, combined with the pinning effect induced by lattice distortion, synergistically reduces the thermal expansion coefficient. In O‐SOFCs, a symmetric cell with GPBSCF exhibits a low polarization resistance of 0.08 Ω cm^2^ at 650°C and operates stably for 1000 h. In R‐PCCs, a single cell demonstrates excellent durability over 680 h. This work provides fundamental insights into high‐entropy optimization mechanisms, guiding the rational design of advanced Fe‐based perovskite air electrodes for durable ceramic electrochemical cells.

## Graphical abstract

A high‐entropy air electrode material, Gd_0.2_Pr_0.2_Ba_0.2_Sr_0.2_Ca_0.2_FeO_3−δ_ (GPBSCF), is developed for both solid oxide fuel cells and reversible protonic ceramic cells. Its enhanced activity stems from increased oxygen vacancies/holes. The stability improvement is attributed to lattice distortion, while thermal expansion reduction is synergistically due to both elevated Fe^4+^ content and lattice distortion.

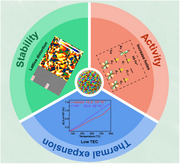

## 1 Introduction

The escalating global energy crisis and environmental pollution have intensified the demand for renewable energy solutions [1]. In this context, ceramic electrochemical cells, particularly oxygen‐ion conducting solid oxide fuel cells (O‐SOFCs) and reversible protonic ceramic cells (R‐PCCs), have emerged as promising technologies for highly efficient interconversion of electrical and chemical energy [2, 3]. A technical challenge that restricts the commercial development of O‐SOFCs and R‐PCCs is the insufficient electrocatalytic activity of the air electrode in the oxygen reduction reaction (ORR) and oxygen evolution reaction (OER) at low temperatures [4]. This issue is further compounded in R‐PCCs, where the electrolysis mode necessitates superior stability of the air electrode against steam. Consequently, it is essential to develop a durable air electrode material with high catalytic activity that is applicable to both O‐SOFCs and R‐PCCs, as this can not only significantly reduce material research and development costs but also effectively simplify the cell manufacturing process.

The current landscape of mixed ion and electron conducting (MIEC) materials, such as La_0.4_Sr_0.6_Co_0.2_Fe_0.8_O_3−δ_ (LSCF) [5], Ba_0.5_Sr_0.5_Co_0.8_Fe_0.2_O_3−δ_ (BSCF) [6], SrCo_0.9_Nb_0.1_O_3−δ_ (SCN) [7], and PrBaCo_2_O_5+𝛿_ (PBC) [8], demonstrates their suitability as air electrodes for O‐SOFCs. Despite their high catalytic activity in O‐SOFCs, these materials exhibit limited proton conductivity and poor steam tolerance, rendering them suboptimal for R‐PCCs [9, 10]. Other typical triple‐conducting (H^+^/O^2−^/e^−^) perovskites are commonly employed in R‐PCCs, such as PrBa_0.5_Sr_0.5_Co_1.5_Fe_0.5_O_5+𝛿_ (PBSCF) [11], BaCo_0.4_Fe_0.4_Zr_0.1_Y_0.1_O_3−δ_ (BCFZY) [12], and BaCo_0.7_(Ce_0.8_Y_0.2_)_0.3_O_3−δ_ (BCCY) [13]. However, the presence of Ba and Sr in these compositions promotes elemental segregation under prolonged steam exposure during electrolysis, leading to perovskite structure degradation and catalytic deactivation [14]. To circumvent Ba/Sr‐related instability, Ding et al. developed a Ba‐ and Sr‐free triple‐conducting material, PrNi_0.5_Co_0.5_O_3−δ_ (PNC), which exhibits remarkable hydration capacity [15]. While Co‐based perovskites generally demonstrate high catalytic activity and rapid ion transport, their high thermal expansion coefficients (TECs), poor thermal compatibility with common electrolytes, elevated fabrication costs, and susceptibility to Co ion segregation under steam limit their practical utility [2, 16, 17]. Fe‐based perovskites offer a promising alternative due to their lower TECs, superior chemical stability, and cost‐effectiveness. However, their catalytic activity remains considerably lower than that of Co‐based analogues. For example, La_0.5_Sr_0.5_FeO_3−δ_ (LSF) exhibits an area‐specific resistance (ASR) approximately 1.68 times higher than La_0.5_Sr_0.5_CoO_3−δ_ (LSC) [18]. To enhance the catalytic performance of Fe‐based perovskites, strategies such as A‐site doping with divalent alkaline earth (Ba^2+^, Sr^2+^, and Ca^2+^) or monovalent alkali metal cations (K^+^, Cs^+^) have been explored. These dopants promote oxygen vacancy formation, thereby improving oxygen reaction kinetics [19, 20]. Nevertheless, such doping often exacerbates cation segregation issues. Thus, achieving a balance between high catalytic activity and long‐term structural stability remains a critical challenge in the development of advanced air electrode materials.

In recent years, high‐entropy strategies have emerged as a promising avenue to address the above challenges [21]. The flexible structural framework of perovskite oxides allows for the incorporation of diverse metal cations at either the A‐ or B‐site, which has significantly accelerated the development of high‐entropy perovskite oxides (HEPOs). The conformational entropy (S_config_) of HEPOs can be determined by the following equation:

$$\begin{array}{ccc} S_{config} & = & -R\left[{\left(\sum_{a=1}^{A}x_{a}lnx_{a}\right)}_{A-\mathrm{site}}+{\left(\sum_{b=1}^{B}x_{b}lnx_{b}\right)}_{B-\mathrm{site}}\right.\\ & & \left.+\;{\left(\sum_{o=1}^{O}x_{o}lnx_{o}\right)}_{O-\mathrm{site}}\right] \end{array}$$

where A/B/O are the number of components at different sites in the perovskite oxide, R is the ideal gas constant, and *x*_*a*/*b*/*o*_ represents the molar fraction of the corresponding element [22]. Based on the S_config_ value, the materials can be categorized into high‐entropy (*S_config_* ≥ 1.5R), medium‐entropy (1R≤ *S_config_* < 1.5R), and low‐entropy (*S_config_* < 1R) materials [23]. Due to the cocktail effect, lattice distortion effect, and sluggish diffusion effect, HEPOs are able to better regulate the structural stability and catalytic activity [24, 25]. HEPOs have been increasingly investigated as air electrodes for both O‐SOFCs and R‐PCCs. Several B‐site HEPOs, such as La_1−x_Sr_x_(Co,Cr,Fe,Mn,Ni)O_3−δ_ and LaMn_0.2_Fe_0.2_Co_0.2_Ni_0.2_Cu_0.2_O_3−δ_, have demonstrated enhanced ORR activity and structural stability [26, 27]. However, as noted by Liu et al., B‐site entropy engineering, while improving structural stability, may partially compromise electrocatalytic performance [28]. This trade‐off arises because the B‐site in perovskites typically hosts catalytically active transition metal cations, and the introduction of certain dopants can markedly alter the electrochemical behavior. Overlooking the intrinsic properties of individual dopants may thus lead to an oversimplified interpretation of the relationship between configurational entropy and electrochemical performance.

To better elucidate the role of configurational entropy, researchers have turned to A‐site high‐entropy design. For instance, He et al. developed Pr_0.2_Ba_0.2_Sr_0.2_La_0.2_Ca_0.2_CoO_3−δ_ (HE‐PBSLCC), which exhibited improved oxygen surface exchange kinetics and operational durability as an air electrode in reversible solid oxide electrochemical cells (R‐SOECs) [29]. When applied in R‐PCCs, HE‐PBSLCC delivered stable performance in both fuel cell (FC) and electrolysis cell (EC) modes, sustaining operation for over 270 h and 500 h, respectively [30]. Nevertheless, the A‐site entropy strategy did not mitigate the high TEC inherent to Co‐based perovskites. The TEC of HE‐PBSLCC (23.8 × 10^−6^ K^−1^) remains substantially higher than that of the BaZr_0.1_Ce_0.7_Y_0.1_Yb_0.1_O_3−δ_ （BZCYYb ） electrolyte (10.2 × 10^−6^ K^−1^) [30]. A‐site high entropy engineering based on Fe‐based perovskites can effectively solve the problem of high TECs. Shang et al. synthesized a low‐TEC Ba_0.2_Sr_0.2_La_0.2_Pr_0.2_Sm_0.2_FeO_3−δ_ (H‐LSF), which exhibited superior electrocatalytic activity compared to its parent La_0.5_Sr_0.5_FeO_3−δ_ (LSF) when applied to O‐SOFCs [31]. Han et al. developed La_0.2_Pr_0.2_Nd_0.2_Sm_0.2_Gd_0.2_BaFe_2_O_5+δ_ (LPNSGBF), which demonstrated high catalytic activity and Cr tolerance in O‐SOFCs [32]. While promising in O‐SOFCs, the performance of these Fe‐based HEPOs in R‐PCCs, especially regarding key metrics such as hydration capacity and steam tolerance, remains to be critically evaluated.

**FIGURE 1:**
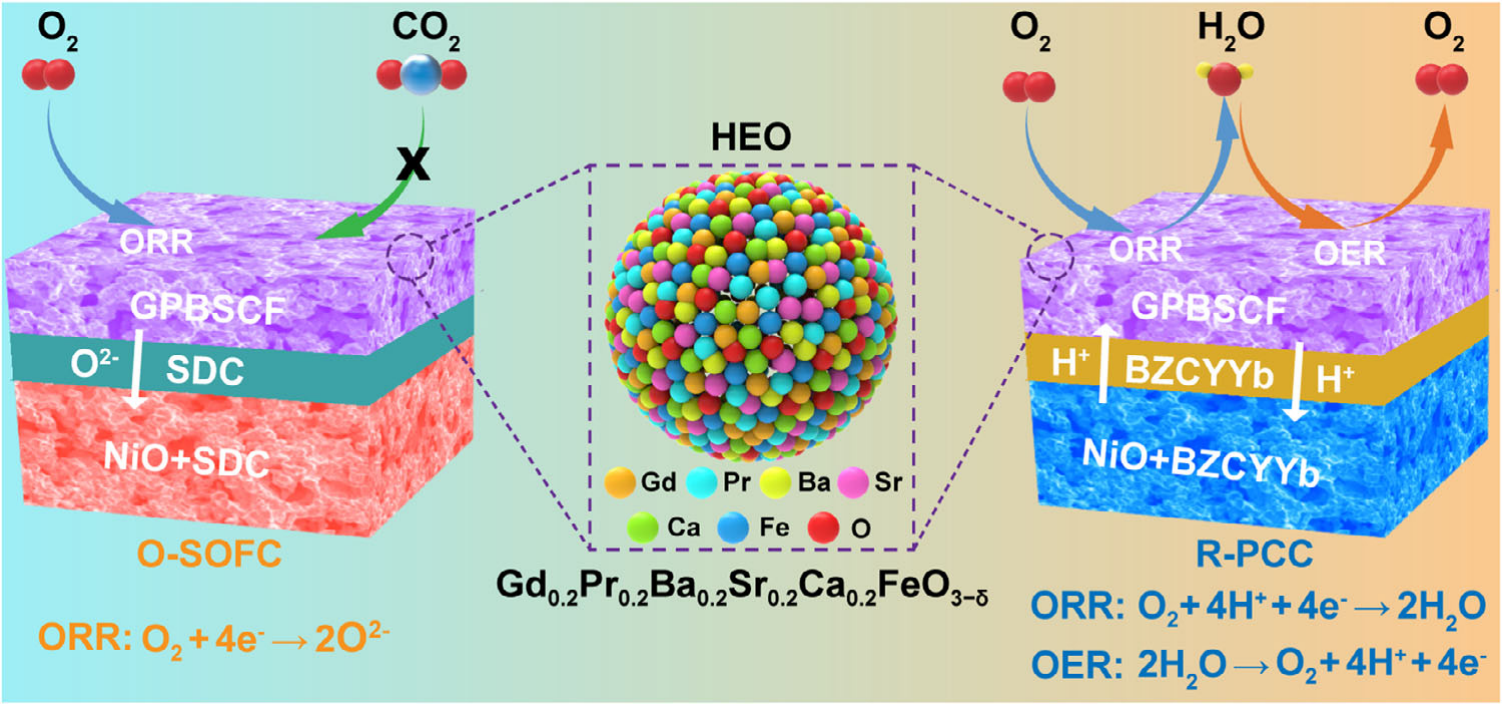
Schematic illustration of O‐SOFCs and R‐PCCs with GPBSCF air electrode.

Although high‐entropy engineering has been employed in ceramic electrochemical cells, its intrinsic effects on activity and stability remain unclear due to a lack of direct evidence. While GdBaFe_2_O_5+δ_ (GBF) has been established as an effective air electrode for O‐SOFCs in prior studies, its potential for R‐PCCs remains unexplored [33, 34]. Herein, we not only assess the feasibility of GBF in R‐PCCs but also employ it as a parent compound to develop an A‐site high‐entropy perovskite, Gd_0.2_Pr_0.2_Ba_0.2_Sr_0.2_Ca_0.2_FeO_3−δ_ (GPBSCF), for application in both O‐SOFCs and R‐PCCs (Figure 1). The GPBSCF air electrode demonstrates several superior attributes, including enhanced electrocatalytic activity, improved structural stability, exceptional tolerance to H_2_O and CO_2_, and impressively low TEC. In O‐SOFC configuration, a symmetric cell withSm_0.2_Ce_0.8_O_1.9_ （SDC ） electrolyte achieved an ASR of 0.08 Ω cm^2^ at 650°C, and a single cell delivered a high peak power density (PPD) of 1.08 W cm^−2^. When applied in R‐PCCs, the GPBSCF electrode exhibited good stability in fuel cell (FC) and electrolysis cell (EC) reversible cycling modes for 100 h. Combined computational and experimental analyses reveal that the high‐entropy doping effectively suppresses the segregation of Ba/Sr species, thereby ensuring structural stability and resistance to H_2_O/CO_2_, while simultaneously increasing the concentration of oxygen vacancies/holes, which consequently enhances the catalytic activity.

## 2 Results and Discussion

**FIGURE 2:**
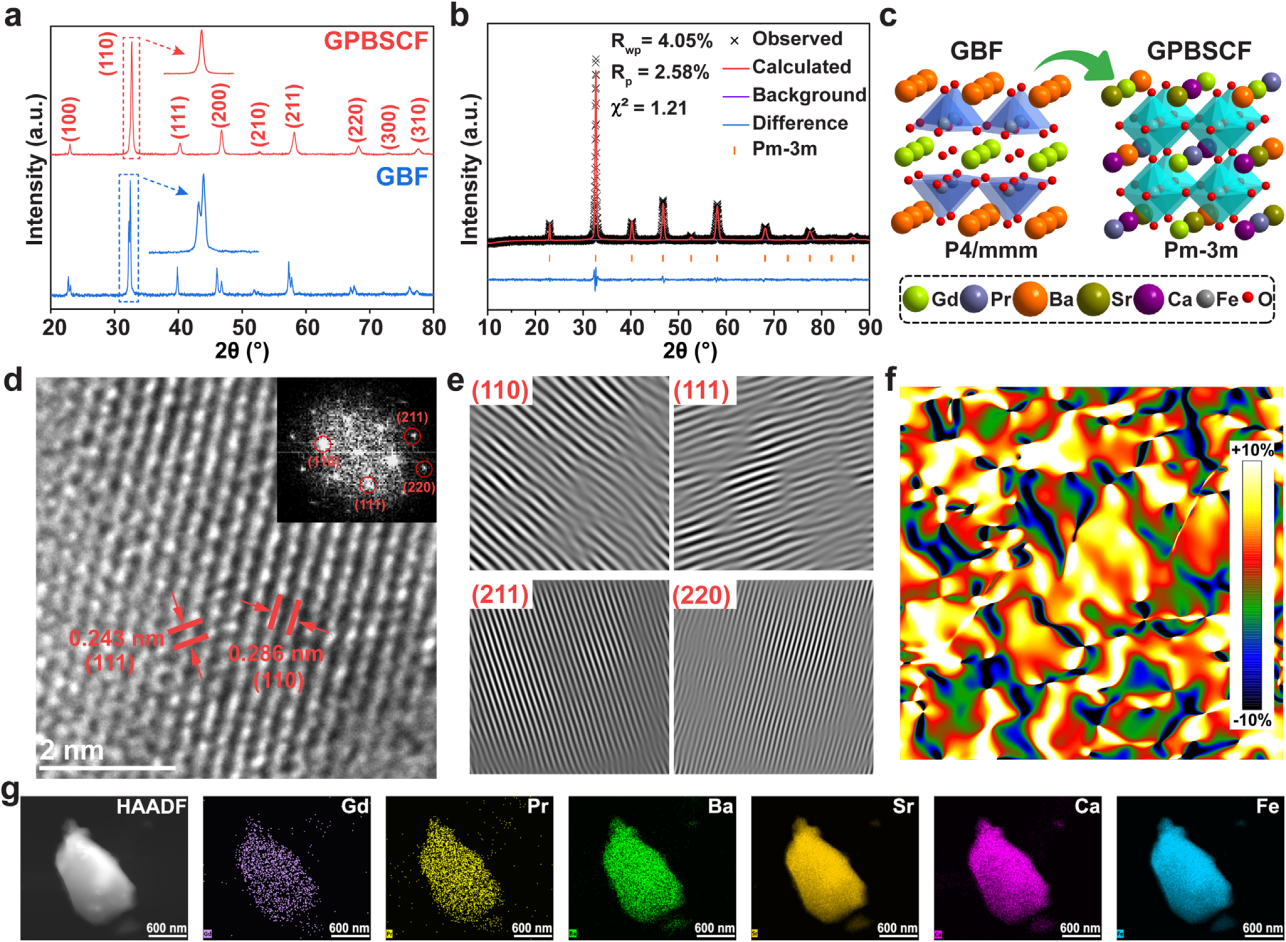
Phase and morphology characterizations. (a) XRD patterns of GBF and GPBSCF powders with a partial enlargement. (b) Refined XRD profile of GPBSCF sample. (c) Schematic illustration of the crystal structure of GBF and GPBSCF. (d) HR‐TEM image of GPBSCF sample. (e) Inverse fast Fourier transform images from HR‐TEM image. (f) GPA strain map of Ɛ_yy_. (g) HAADF‐STEM images and corresponding EDS mapping of GPBSCF sample.

The phase structures of the as‐prepared GBF and GPBSCF were examined by room‐temperature X‐ray diffraction (XRD), as shown in Figure 2a. Both the parent double perovskite GBF and the high‐entropy perovskite GPBSCF exhibit single‐phase structures without any impurities. XRD Rietveld refinement results show that GPBSCF exhibits a cubic perovskite structure with a space group of *Pm*‐3*m*, whereas GBF adopts a tetragonal structure with a space group of *P*4/*mmm*, as shown in Figure 2b and Figure S1. The corresponding unit cell parameters are summarized in Table S1. A schematic illustration of the crystal structures visually depicts the phase transformation from tetragonal (GBF) to cubic (GPBSCF) induced by the high‐entropy strategy (Figure 2c). This transition to a higher symmetry structure is particularly advantageous, as cubic perovskites are generally associated with superior oxygen ion and proton transport properties [35]. The transition from a lower‐symmetry tetragonal phase to a cubic structure reduces the O–O and Metal–O bond lengths, thereby shortening the migration pathways for oxygen ions and protons. This reduction in conduction distance facilitates ion mobility and lowers the associated energy barrier [36]. Additionally, the low‐symmetry phase generates unequal oxygen sites that act as isolated low‐energy trapping sites, which further suppresses the conduction of both protons and oxygen ions [37]. The structural stability of GPBSCF at operating temperature was explored by in situ high‐temperature XRD (HT‐XRD). As shown in Figure S2, no secondary phases are detected during heating from room temperature to 800°C, confirming the excellent phase stability of GPBSCF within this temperature range. The chemical compatibility between GPBSCF and SDC/BZCYYb electrolyte also needs to be evaluated. As shown in Figure S3, GPBSCF and SDC/BZCYYb electrolytes have good chemical compatibility without new phase generation after high‐temperature calcination. Further structural analysis was performed using high‐resolution transmission electron microscopy (HR‐TEM). Figure 2d reveals well‐defined lattice fringes with interplanar spacings of 0.286 and 0.243 nm, corresponding to the (110) plane and (111) plane of GPBSCF, respectively. Similarly, the structure of GBF was characterized. As shown in Figure S4a, the lattice spacings corresponding to the (100) and (200) crystal planes in GBF are measured as 0.408 and 0.203 nm, respectively. The inverse fast Fourier transform (IFFT) images of GPBSCF and GBF are presented in Figure 2e and Figure S4b. The results clearly show that the lattice fringes of GBF, after IFFT processing, are well‐aligned and regular, with no observable distortion. In contrast, the IFFT‐processed lattice fringes of GPBSCF exhibit significant lattice distortion and discontinuity. This indicates the presence of severe lattice distortion within GPBSCF, primarily attributed to the high‐entropy doping at the A‐site with multiple cations of mismatched ionic radii, which induces substantial local lattice distortion and stress fields [1]. The lattice distortion in GPBSCF was further validated using the geometric phase analysis (GPA) method. Figure 2f reveals pronounced strain along the Ɛ_yy_ direction, manifested as numerous discontinuous white and black regions [1]. In addition, High‐angle annular dark‐field scanning transmission electron microscopy (HAADF‐STEM) and energy dispersive spectrometer (EDS) mapping confirm a homogeneous nanoscale distribution of all constituent elements in GPBSCF (Figure 2g). Collectively, these structural analyses verify the successful synthesis of the A‐site high‐entropy perovskite GPBSCF and underscore its robust structural integrity and chemical compatibility.

**FIGURE 3:**
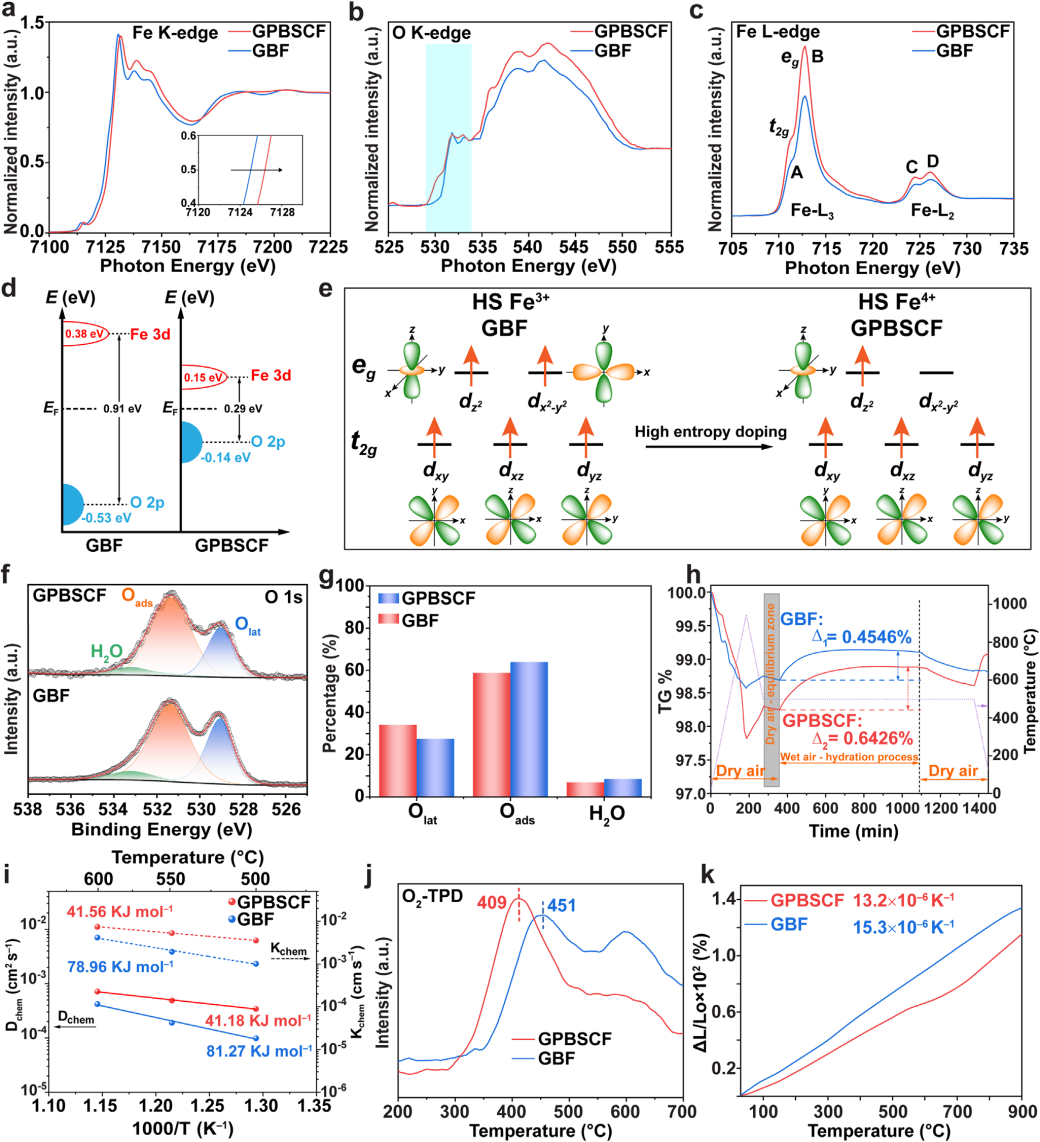
Material properties characterizations. (a) Fe K‐edge XANES and (b) O K‐edge XANES and (c) Fe L‐edge XANES and (d) the DFT‐calculated band center and charge transfer energy and (e) Illustration of the 3d electron configuration of Fe species and (f) the fitted XPS spectra of O 1s and (g) comparisons of O 1s and (h) TGA curves and (i) the fitted D_chem_ and K_chem_ values and (j) O_2_‐TPD profiles and (k) TECs for GBF and GPBSCF.

Excellent electrocatalytic activity for both the ORR and OER is a key requirement for ideal air electrode materials, strongly influenced by the valence state of the B‐site transition metal and the surface oxygen vacancy concentration [38]. To probe the electronic structure and Fe valence state in the perovskite samples, X‐ray absorption fine structure (XAFS) analysis was conducted on Fe, Gd, and O in both GBF and GPBSCF. As shown in Figure 3a, the Fe K‐edge X‐ray absorption near‐edge structure (XANES) spectra reveal a slightly higher average valence of Fe in GPBSCF compared to GBF. This finding is corroborated by X‐ray photoelectron spectroscopy (XPS) results (Figure S6 and Table S2), which also indicate an increased Fe oxidation state in the high‐entropy material. The Gd oxidation state, in contrast, remains consistent in both perovskites (Figure S5). The elevation in Fe valence is likely attributed to the incorporation of lower‐valent alkaline‐earth metal cations at the A‐site, which promotes charge compensation [20]. A higher Fe^4+^ content is favorable, as it increases hole concentration and enhances electronic conductivity [39]. Consistent with this, the GPBSCF sample exhibits significantly higher electronic conductivity than GBF (Figure S7). This improvement can be ascribed not only to the increased Fe^4+^ content but also to the highly symmetric cubic perovskite structure, which facilitates electron transport. The O K‐edge XANES spectra were normalized within the range of 525–555 eV (Figure 3b). The peak in the 529–534 eV region (shaded area) corresponds to the O 2p orbitals hybridized with the Fe 3d orbitals, which are split into t_2g_ (d_xy_, d_xz_, d_yz_) and e_g_ (d_z_^2^, d_x_^2^_–y_^2^) orbitals under the octahedral crystal field. The integrated area under this curve is indicative of the corresponding oxygen hole states. A larger integrated area signifies a higher concentration of oxygen holes. The significantly larger area observed for GPBSCF, compared to that of GBF, indicates a higher oxygen hole concentration and hence better redox activity [40]. Figure 3c displays the normalized Fe L‐edge XANES spectra in the energy range of 705–735 eV. Peaks A and C correspond to the Fe 3d t_2g_ orbitals, while peaks B and D are associated with the Fe 3d e_g_ orbitals [20]. The intensity ratio of peak B to peak A is 2.06 in GBF, whereas this value in GPBSCF is 2.08. The higher ratio suggests a greater number of vacant e_g_ orbitals in GPBSCF, which is attributed to its higher proportion of high‐spin Fe^4+^ species. To further investigate the orbital hybridization between O 2p and Fe 3d in the two samples, density functional theory (DFT) calculations were employed to determine the partial density of states (PDOS) of the O 2p and Fe 3d orbitals (Figure S8). As shown in Figure 3d, the charge transfer energy, defined as the energy difference between the centers of the O 2p and Fe 3d bands, is closely related to the oxygen surface exchange kinetics and the ORR/OER activity [40]. Compared to the parent GBF, high‐entropy doping induces a shift of the O 2p band center in GPBSCF closer to the Fermi level, reducing the charge transfer energy from 0.91 to 0.29 eV. This indicates a lower electronic transfer barrier and enhanced catalytic reaction kinetics in GPBSCF. As shown in Figure 3e, compared to GBF, the high‐entropy doping in GPBSCF results in a higher proportion of Fe^4+^ and a greater concentration of oxygen holes, thereby enhancing the catalytic activity of the electrode material.

Figure 3f displays the deconvoluted O 1s XPS spectra of the GBF and GPBSCF samples. Based on binding energy, the spectra can be resolved into three distinct peaks, corresponding to lattice oxygen (O_lat_ ≈ 529 eV), surface‐adsorbed oxygen (O_ads_ ≈ 531 eV), and adsorbed H_2_O (H_2_O ≈ 533 eV) [3]. As summarized in Figure 3g and Table S3, GPBSCF exhibits higher proportions of both adsorbed oxygen and H_2_O relative to GBF. The increased O_ads_/O_lat_ ratio in GPBSCF suggests a higher concentration of surface oxygen vacancies, while the elevated adsorbed H_2_O content implies enhanced hydration capability compared to GBF [41]. These findings are further supported by thermogravimetric (TG) analysis conducted in wet air. As illustrated in Figure 3h, the samples were first heated to 950°C in dry air to remove physisorbed H_2_O, followed by isothermal holding at 500°C for 12 h under wet air (3% H_2_O) to allow hydration. The weight gains of GBF and GPBSCF were 0.45% and 0.64%, respectively, confirming the superior hydration capacity of GPBSCF and its suitability as an air electrode for R‐PCCs.

The electrocatalytic activity of electrode materials is closely linked to their oxygen surface exchange and bulk diffusion properties. Electrical conductivity relaxation (ECR) measurements were performed by abruptly changing the oxygen partial pressure from 0.21 to 0.10 atm (Figure S9). As shown in Figure 3i and Table S4, GPBSCF exhibits higher surface exchange coefficients (K_chem_) and bulk diffusion coefficients (D_chem_) than GBF, indicating that A‐site high‐entropy engineering facilitates both oxygen surface exchange and bulk transport, thereby accelerating ORR/OER kinetics. The reliability of these results was further verified by O_2_ temperature programmed desorption (O_2_‐TPD). After pretreatment in helium to remove surface‐adsorbed oxygen, the desorption peaks in Figure 3j originate primarily from lattice oxygen. The noticeably lower desorption temperature for GPBSCF indicates enhanced mobility of oxygen ions, consistent with its improved oxygen transport kinetics [42].

The long‐term operational stability of ceramic electrochemical cells is critically dependent on the thermal expansion compatibility between the air electrode and the electrolyte. Figure 3k displays the thermal expansion curves of GBF and GPBSCF recorded in air from 30°C to 900°C. The TECs for GBF and GPBSCF are 15.3 × 10^−6^ K^−1^ and 13.2 × 10^−6^ K^−1^, respectively. Compared to the parent GBF, the pronounced lattice distortion in GPBSCF generates a pinning effect that suppresses atomic vibrations, while the higher Fe^4+^ content inhibits chemical expansion caused by the reduction of transition metal ions. This result clearly demonstrates that the A‐site high‐entropy strategy effectively reduces the TEC of the material, which is expected to enhance the interfacial adhesion and thermomechanical compatibility with the electrolyte at elevated temperatures. Notably, the TEC of GPBSCF is substantially lower than those of typical Co‐based air electrodes, such as HE‐PBSLCC (23.8 × 10^−6^ K^−1^) and (La_0.25_Pr_0.25_Nd_0.25_Sm_0.25_)Ba_0.5_Sr_0.5_Co_1.5_Fe_0.5_O_5+δ_ (18.93 × 10^−6^ K^−1^) [30, 43].

**FIGURE 4:**
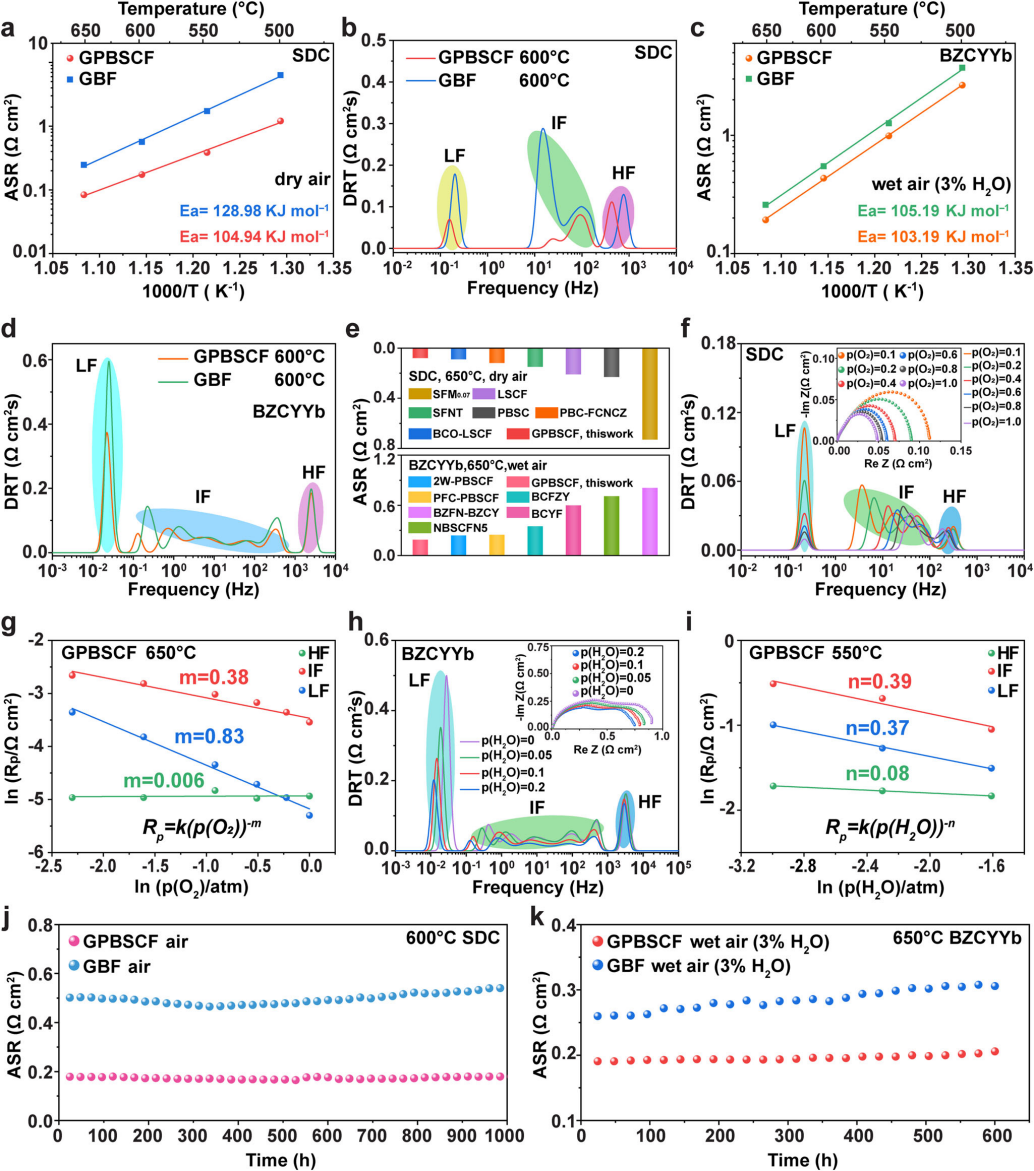
Electrochemical performance and durability of the GBF and GPBSCF electrodes. Temperature dependence of the ASRs of GBF and GPBSCF electrodes with (a) SDC‐based and (c) BZCYYb‐based symmetric cells. DRT plots of EIS of GBF and GPBSCF electrodes with (b) SDC‐based and (d) BZCYYb‐based symmetric cells at 600°C. (e) ASR comparison of the GPBSCF and other reported electrodes at 650°C. (f) DRT of GPBSCF electrode as a function of *p*_O2_ measured at 650°C; inset is the corresponding EIS. (g) Dependence of each *R*_p_ at HF, IF, and LF ranges as a function of *p*_O2_. (h) DRT of GPBSCF electrode as a function of *p*_H2O_ measured at 550°C; inset is the corresponding EIS. (i) Dependence of each *R*_p_ at HF, IF, and LF ranges as a function of *p*_H2O_. Time dependence of ASRs of GPBSCF electrode with (j) SDC‐based symmetric cells in air at 600°C and (k) BZCYYb‐based symmetric cells in wet air at 650°C.

The electrochemical performance and durability of GBF and GPBSCF electrodes were evaluated by measuring the ASR (*R*_p_) of symmetric cells fabricated with SDC and BZCYYb electrolytes. As shown in Figure 4a, the ASR values of the GPBSCF electrode on SDC electrolyte at 650°C, 600°C, 550°C, and 500°C are 0.08, 0.17, 0.39, and 1.21 Ω cm^2^, respectively, significantly lower than those of GBF (0.25, 0.57, 1.73, and 6.33 Ω cm^2^), indicating superior ORR catalytic activity for GPBSCF. The corresponding electrochemical impedance spectra (EIS) are provided in Figure S10. To gain further insight into the effect of A‐site high‐entropy design on electrocatalytic activity, the distribution of relaxation times (DRT) method was employed to deconvolute the EIS data into constituent electrochemical processes, thereby identifying the rate‐limiting steps for ORR [44]. The DRT spectra can be partitioned into three regions: the high‐frequency (HF) region, associated with ion or charge transfer at the electrode‐electrolyte interface; the intermediate‐frequency (IF) region, related to ion transport within the electrode or surface exchange; and the low‐frequency (LF) region, reflecting gas adsorption and diffusion processes [45, 46]. As shown in Figure 4b, the DRT curve of the GPBSCF electrode on SDC at 600°C exhibits a substantially reduced IF peak compared to GBF, suggesting enhanced oxygen ion transport kinetics. This observation aligns well with the XPS and ECR results. The marked optimization of the IF part also contributes to the reduction in the HF and LF regions. Similarly, when tested on a BZCYYb electrolyte, the GPBSCF electrode demonstrates lower ASR values (0.19, 0.44, 0.99, and 2.66 Ω cm^2^) than GBF (0.26, 0.55, 1.27, and 3.73 Ω cm^2^) at 650°C, 600°C, 550°C, and 500°C (Figure 4c). The corresponding EIS data are shown in Figure S11. The DRT analysis under this configuration reveals decreased intensities in both the IF and LF regions for GPBSCF, implying improved hydration capability and enhanced proton generation in the bulk phase, consistent with XPS and TG findings (Figure 4d). Cross‐sectional scanning electron microscopy images of the symmetrical cells confirm that both GBF and GPBSCF electrodes exhibit similar porosity, grain size, and thickness, effectively ruling out microstructure‐related variations in polarization resistance (Figures S12 and S13). Furthermore, as summarized in Figure 4e and Tables S5 and S6, the GPBSCF air electrode achieves lower ASR values than most state‐of‐the‐art air electrodes [47, 48, 49, 50, 51, 52, 53, 54, 55, 56, 57].

To further investigate the ORR/OER kinetics of the GPBSCF electrode, the EIS curves and corresponding DRT analysis were performed on symmetrical cells at different oxygen partial pressures (*p*_O2_) and water partial pressures (*p*_H2O_) (Figure 4f,h). The ASR values of the GPBSCF electrode decreased with increasing *p*_O2_ and *p*_H2O_, confirming its triple‐conducting (H^+^/O^2−^/e^−^) nature and validating its suitability as a versatile air electrode for both O‐SOFCs and R‐PCCs. In addition, the relationship between *R*_p_ and *p*_O2_ corresponding to the different processes can be expressed as the equation *R*_p_ = *k*(*p*_O2_)^−m^, where the m value represents the reaction order relative to *p*_O2_ (Figure 4g) [45]. The m values corresponding to the HF, IF, and LF processes are 0.006, 0.38, and 0.83, respectively. This indicates that the electrochemical process in the HF part is largely independent of *p*_O2_, while the reaction processes corresponding to the IF and LF parts are closely related to the external oxygen concentration. Given that the IF process exhibits the highest ASR among all processes, it is identified as the rate‐determining step for the GPBSCF electrode on the SDC electrolyte. Similarly, the influence of *p*_H2O_ was quantified using the relation *R*_p_ = *k*(*p*_H2O_)^−n^, where the value of n represents the reaction order relative to *p*_H2O_ [30]. As shown in Figure 4i, the lower n value in the HF region implies negligible sensitivity to *p*_H2O_, while the IF and LF parts are more sensitive to the external *p*_H2O_. Again, the dominant contribution of the IF region to the total ASR establishes it as the rate‐limiting step for the GPBSCF electrode on the BZCYYb electrolyte.

To verify the effect of A‐site high entropy engineering on electrode durability, the ASR stability of symmetric cells with GBF and GPBSCF electrodes using SDC/BZCYYb as the electrolyte was also evaluated, as shown in Figure 4j,k. The corresponding EIS graphs are shown in Figures S14 and S15. On the SDC electrolyte, the GPBSCF electrode exhibited exceptional stability, demonstrating a negligible ASR degradation rate of 1.0 × 10^−6^ Ω cm^2^ h^−1^ over 1000 h, significantly lower than that of GBF (3.92 × 10^−5^ Ω cm^2^ h^−1^). A similar trend was observed with the BZCYYb electrolyte, where GPBSCF showed a degradation rate of 2.5 × 10^−5^ Ω cm^2^ h^−1^ over 600 h, compared to 7.67 × 10^−5^ Ω cm^2^ h^−1^ for GBF. These results collectively confirm that the A‐site high‐entropy strategy markedly enhances the long‐term operational stability of the electrode.

The reduced TEC also enhances the thermal compatibility between GPBSCF and SDC/BZCYYb, effectively improving the long‐term operational stability of the symmetric cell.

**FIGURE 5:**
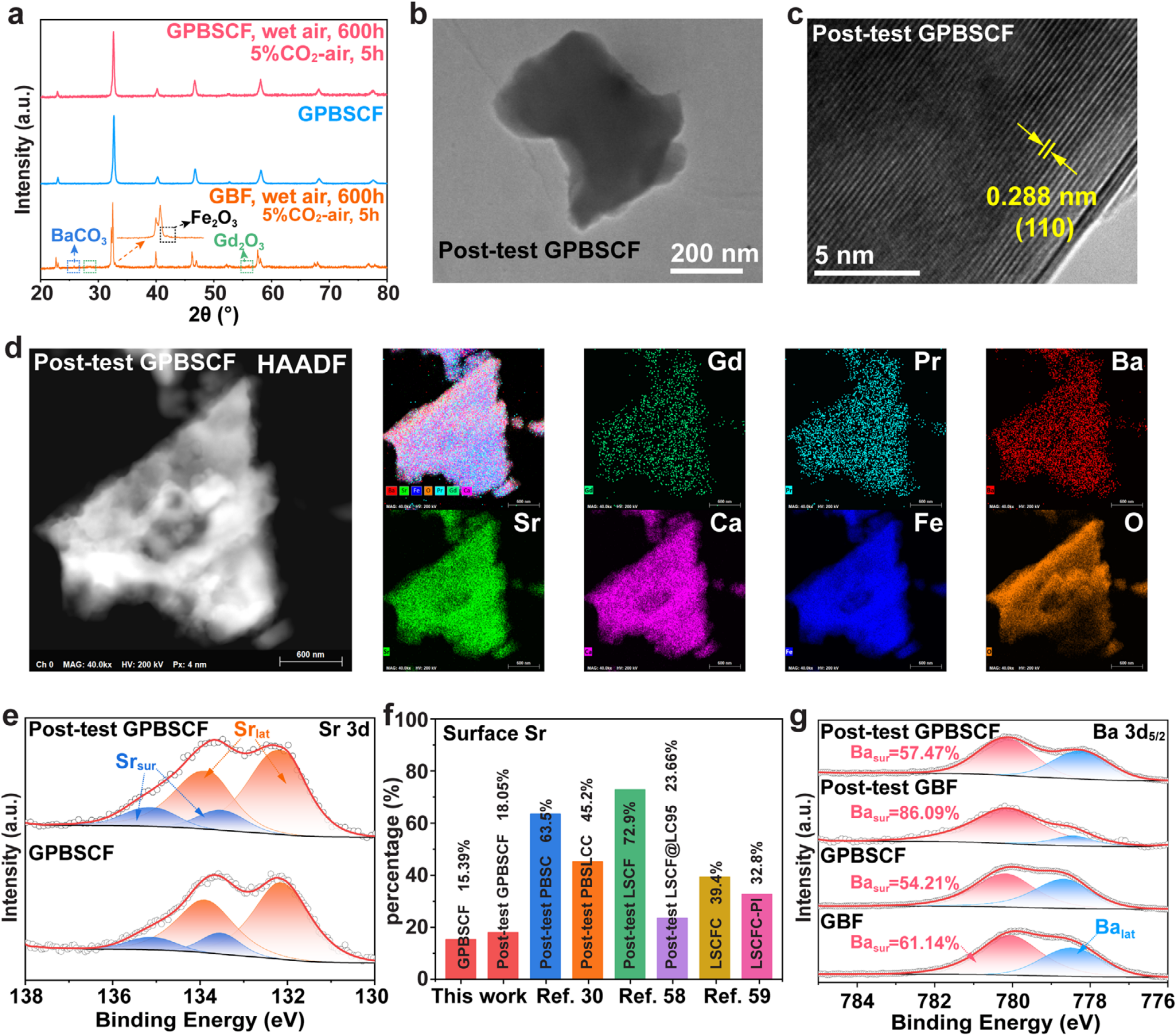
Structural and morphological analysis of GPBSCF after treatment in wet air and CO_2_. (a) XRD patterns of GBF and GPBSCF powders after treatment in wet air for 600 h and in 5% CO_2_‐air for 5 h at 650°C. (b) STEM image, (c) HR‐TEM image, and (d) HAADF‐EDS images of post‐test GPBSCF. (e) Sr 3d XPS curves of GPBSCF and post‐test GPBSCF. (f) Surface Sr contents comparisons of GPBSCF, post‐test GPBSCF, and other reported electrodes. (g) Ba 3d XPS curves of GBF, GPBSCF, post‐test GBF, and post‐test GPBSCF.

To evaluate the tolerance of GBF and GPBSCF electrode materials toward H_2_O and CO_2_, both samples were subjected to a 600 h stability test in wet air, followed by exposure to air containing 5% CO_2_ at 650°C for 5 h. As shown in Figure 5a, the XRD pattern of GPBSCF after this treatment remains free of impurity phases, confirming its excellent chemical stability under harsh operating environments. In contrast, the parent GBF material developed secondary phases under identical conditions, underscoring its unsuitability as an air electrode for R‐PCCs. Post‐test characterization further validates the structural integrity of GPBSCF. The STEM image reveals a smooth surface without nanoparticle precipitation or steam‐induced phase separation (Figure 5b). HR‐TEM analysis identifies clear lattice fringes with a spacing of 0.288 nm, corresponding to the (110) plane, which closely matches the value of 0.286 nm obtained prior to the stability test (Figure 5c). HAADF‐STEM and EDS mapping further confirm a homogeneous elemental distribution without agglomeration after prolonged operation (Figure 5d). XPS analysis was employed to monitor surface segregation behavior. The Sr 3d spectra demonstrate no significant increase in surface Sr content on GPBSCF after the stability test (Figure 5e). As shown in Figure 5f, GPBSCF exhibits a superior capability to suppress surface Sr segregation compared to other recently reported state‐of‐the‐art air electrode materials [30, 58, 59]. Similarly, Ba 3d spectra show a significant increase in surface Ba content for GBF after stability test (from 61.14% to 86.09%), while GPBSCF exhibit only a slight change (from 54.21% to 57.47%) (Figure 5g) [60]. The above results indicate that high‐entropy doping effectively suppresses surface segregation of Ba and Sr elements. In addition, as shown in Figure S16, Fourier transform infrared spectroscopy (FTIR) confirms the persistence of the ${\left(OH\right)}_{o}^{\cdot }$ characteristic peak in GPBSCF even after 600 h of stability test [61].

**FIGURE 6:**
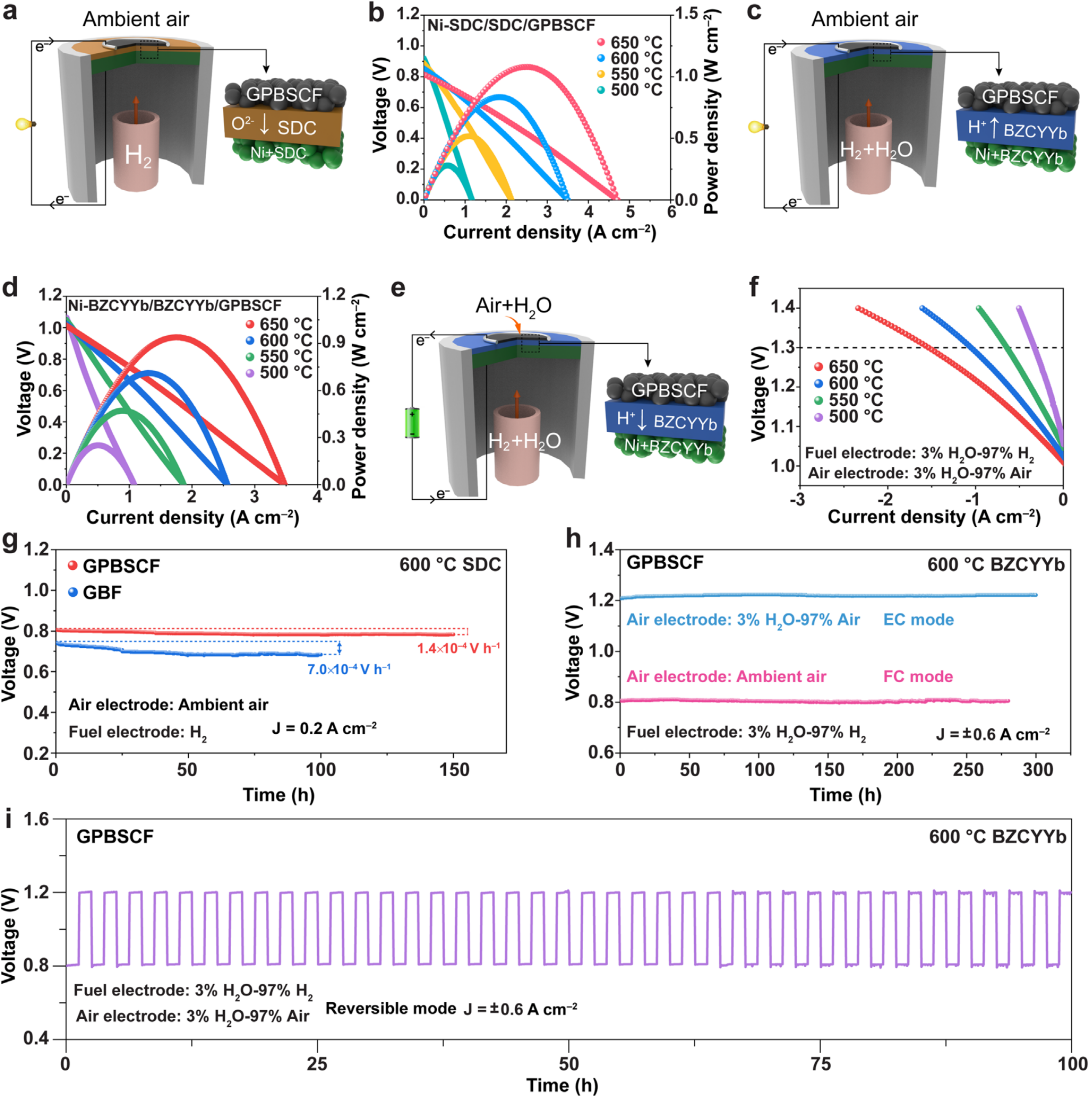
Application evaluation of O‐SOFCs and R‐PCCs with GPBSCF air electrode. (a) An illustration of a SDC‐based anode‐supported single cell. (b) *I*‐*V*‐*P* curves of a SDC‐based single cell with a GPBSCF electrode. (c) An illustration of a BZCYYb‐based anode‐supported single cell. (d) *I*‐*V*‐*P* curves of a BZCYYb‐based single cell with a GPBSCF electrode in the FC mode. (e) An illustration of a BZCYYb‐based anode‐supported electrolysis cell. (f) *I*‐*V* curves of a BZCYYb‐based single cell with a GPBSCF electrode in the EC mode. Stability test of (g) the SDC‐based single cells and a constant current density of 0.2 A cm^−2^ with GBF and GPBSCF electrodes and (h) the BZCYYb‐based single cells and a constant current density of 0.6 A cm^−2^(FC mode) and −0.6 A cm^−2^(EC mode) with a GPBSCF electrode at 600°C. (i) Stability test of reversible operation of a BZCYYb‐based cell with GPBSCF electrode cycling between FC and EC modes.

The electrochemical performance of the GPBSCF air electrode was evaluated in anode‐supported single cells with the configurations Ni‐SDC|SDC|GPBSCF for O‐SOFCs and Ni‐BZCYYb|BZCYYb|GPBSCF for R‐PCCs (Figure 6a,c,e). Figure 6b and Figure S17 show the current‐voltage‐power (*I*‐*V*‐*P*) curves of the SDC‐based single cells with GPBSCF and GBF air electrodes. At 650°C, 600°C, 550°C, and 500°C, the PPD values of the single cell with GPBSCF electrode are 1.08, 0.84, 0.52, and 0.28 W cm^−2^, respectively, which are much higher than those of the GBF‐based cell. The comparable electrolyte thickness in both cells ensures a fair comparison of the electrocatalytic activity between the GBF and GPBSCF electrodes (Figure S18). Under open circuit voltage (OCV) conditions, the GPBSCF electrode demonstrated markedly lower ASR values than GBF, confirming its superior electrocatalytic activity (Figure S19). Furthermore, as summarized in Figure S20 and Table S7, the GPBSCF electrode outperforms most reported air electrodes in O‐SOFC operation. A similar trend was observed for BZCYYb‐based single cells in FC mode (Figure 6d and Figure S21). The GPBSCF‐based cell achieved PPD values of 0.94, 0.71, 0.47, and 0.25 W cm^−2^ at 650°C–500°C, surpassing the performance of the GBF‐based cell (Table S8). The two BZCYYb‐based single cells also have the same electrolyte thickness (Figure S22). As shown in Figure S23, in the BZCYYb‐based single cell, the GPBSCF electrode also exhibits lower ASR values, which indicates that the GPBSCF electrode has higher ORR catalytic activity. The OER catalytic activity of the GPBSCF electrode was further evaluated in EC mode by feeding wet air (3% H_2_O) to the air electrode. Figure 6f and Figure S24 show the *I*‐*V* curves of the BZCYYb‐based single cells with GBF and GPBSCF air electrodes in EC mode. At 650°C, 600°C, 550°C, and 500°C, the electrolysis current densities of the GPBSCF electrode at 1.3 V were 1.54, 1.05, 0.64, and 0.33 A cm^−2^, respectively, which were higher than those of the GBF electrode (Table S9). Notably, the GPBSCF electrode outperforms most advanced air electrodes in both FC and EC modes, as shown in Figure S25.

In addition, the long‐term operational stability of both types of single cells was systematically evaluated. For O‐SOFCs, the SDC‐based single cell with the GPBSCF electrode exhibited superior stability, with a voltage degradation rate of merely 1.4 × 10^−4 ^V h^−1^, compared to a significantly higher decay rate of 7 × 10^−4 ^V h^−1^ for the GBF‐based cell (Figure 6g). For R‐PCCs, three consecutive tests were conducted on a BZCYYb‐based single cell with the GPBSCF electrode: the cell was first tested in EC mode for 300 h, then switched to FC mode for 280 h, and finally operated in reversible cycling mode for another 100 h, yielding a total stability test duration of 680 h. As shown in Figure 6h, the cell operated at a constant current density of ±0.6 A cm^−2^ at 600°C exhibited excellent durability in both FC and EC modes.  Furthermore, by cyclically switching between FC (+0.6 A cm^−2^) and EC (−0.6 A cm^−2^) modes at 600°C, the GPBSCF electrode demonstrated outstanding reversible operational stability, as illustrated in Figure 6i. Concurrently, Figure S26 confirms the integrity of the interface between the GPBSCF electrode and the BZCYYb electrolyte following the durability test, showing no signs of delamination. The combined results of high output performance and exceptional operational stability across multiple testing modes affirm that GPBSCF is a highly viable air electrode material for both O‐SOFCs and R‐PCCs.

## 3 Conclusion

In summary, we developed a highly active and structurally robust Fe‐based high‐entropy perovskite material GPBSCF by A‐site high‐entropy engineering, which can be used as air electrode for both O‐SOFCs and R‐PCCs. Our research uncovered two intrinsic effects of the high‐entropy doping on the activity and stability of air electrode: the enhanced catalytic activity of the high‐entropy perovskite GPBSCF fundamentally stems from increased concentrations of oxygen vacancies and holes, while its improved structural stability is primarily due to the introduced lattice distortion. Moreover, GPBSCF shows a markedly lower TEC than GBF and Co‐based perovskites, due to the increased Fe^4+^ proportion and lattice distortion that suppress atomic vibrations. Symmetric cells employing the GPBSCF electrode exhibit excellent electrochemical stability for over 1000 and 600 h on SDC and BZCYYb electrolytes, respectively. Notably, a BZCYYb‐based single cell with the GPBSCF electrode maintains outstanding stability during a 680‐h test. Our study elucidates the intrinsic mechanisms of the high‐entropy air electrode, thereby providing valuable insights and practical guidelines for the rational design of air electrodes in ceramic electrochemical cells using the high‐entropy strategy.

## 4 Experimental Section

The details of powder synthesis, cell fabrication, materials characterization, and electrochemical testing are provided in the Supporting Information.

## Funding

This work was financially supported by the National Natural Science Foundation of China (No. 91745203) and the Fundamental Research Funds for the Central Universities.

## Conflicts of Interest

The authors declare no conflicts of interest.

## Supporting information




**Supporting File**: advs75100‐sup‐0001‐SuppMat.docx.

## Data Availability

The data that support the findings of this study are available from the corresponding author upon reasonable request.

## References

[1] W. Zhang , W. Ma , Y. Gao , et al., “Small‐Size Atom‐Driven Distortion Realizes High‐Entropy Oxides With Simultaneous Chemical Stability and Activity Enhancement Enabling a Practical Cathode for Solid Oxide Fuel Cells,” Applied Catalysis B: Environment and Energy 383 (2026): 126147, 10.1016/j.apcatb.2025.126147.

[2] Y. Li , C. Yang , X. Liu , et al., “Structurally Stable Perovskite Cathode With Extended Lifetime for Protonic Ceramic Fuel Cells,” ACS Sustainable Chemistry & Engineering 13, no. 12 (2025): 4740–4749, 10.1021/acssuschemeng.4c09923.

[3] Y. Zhang , Y. Wang , Z. Liu , et al., “Constructing Robust and Efficient Ceramic Cells Air Electrodes through Collaborative Optimization Bulk and Surface Phases,” Advanced Functional Materials 35, no. 20 (2025): 2422531, 10.1002/adfm.202422531.

[4] N. Wang , C. Tang , L. Du , et al., “Advanced Cathode Materials for Protonic Ceramic Fuel Cells: Recent Progress and Future Perspectives,” Advanced Energy Materials 12, no. 34 (2022): 2201882, 10.1002/aenm.202201882.

[5] A. Mai , V. A. C. Haanappel , S. Uhlenbruck , F. Tietz , and D. Stöver , “Ferrite‐Based Perovskites as Cathode Materials for Anode‐Supported Solid Oxide Fuel Cells,” Solid State Ionics 176, no. 15 (2005): 1341–1350, 10.1016/j.ssi.2005.03.009.

[6] Z. Shao and S. M. Haile , “A High‐Performance Cathode for the Next Generation of Solid‐Oxide Fuel Cells,” Nature 431, no. 7005 (2004): 170–173, 10.1038/nature02863. 15356627

[7] K. Zhu , Y. Yang , D. Huan , et al., “Theoretical and Experimental Investigations on K‐doped SrCo_0.9_Nb_0.1_O_3‐δ_ as a Promising Cathode for Proton‐Conducting Solid Oxide Fuel Cells,” Chemsuschem 14, no. 18 (2021): 3876–3886, 10.1002/cssc.202101100. 34265159

[8] G. Kim , S. Wang , A. J. Jacobson , L. Reimus , P. Brodersen , and C. A. Mims , “Rapid Oxygen Ion Diffusion and Surface Exchange Kinetics in PrBaCo_2_O_5+x_ With a Perovskite Related Structure and Ordered A Cations,” Journal of Materials Chemistry 17, no. 24 (2007): 2500–2505, 10.1039/B618345J.

[9] X. Xu , C. Su , and Z. Shao , “Fundamental Understanding and Application of Ba_0.5_Sr_0.5_Co_0.8_Fe_0.2_O^3–δ^ Perovskite in Energy Storage and Conversion: Past, Present, and Future,” Energy & Fuels 35, no. 17 (2021): 13585–13609, 10.1021/acs.energyfuels.1c02111.

[10] H. Shimada , Y. Yamaguchi , H. Sumi , and Y. Mizutani , “Enhanced La_0.6_Sr_0.4_Co_0.2_Fe_0.8_O_3–δ_‐Based Cathode Performance by Modification of BaZr_0.1_Ce_0.7_Y_0.1_Yb_0.1_O_3–δ_ electrolyte Surface in Protonic Ceramic Fuel Cells,” Ceramics International 47, no. 11 (2021): 16358–16362, 10.1016/j.ceramint.2021.02.123.

[11] S. Choi , C. J. Kucharczyk , Y. Liang , et al., “Exceptional Power Density and Stability at Intermediate Temperatures in Protonic Ceramic Fuel Cells,” Nature Energy 3, no. 3 (2018): 202–210, 10.1038/s41560-017-0085-9.

[12] X. Kuai , G. Yang , Y. Chen , et al., “Boosting the Activity of BaCo_0.4_Fe_0.4_Zr_0.1_Y_0.1_O_3−δ_ Perovskite for Oxygen Reduction Reactions at Low‐to‐Intermediate Temperatures Through Tuning B‐Site Cation Deficiency,” Advanced Energy Materials 9, no. 38 (2019): 1902384, 10.1002/aenm.201902384.

[13] Y. Song , Y. Chen , W. Wang , et al., “Self‐Assembled Triple‐Conducting Nanocomposite as a Superior Protonic Ceramic Fuel Cell Cathode,” Joule 3, no. 11 (2019): 2842–2853, 10.1016/j.joule.2019.07.004.

[14] M. Choi , S. J. Kim , and W. Lee , “Effects of Water Atmosphere on Chemical Degradation of PrBa_0.5_Sr_0.5_Co_1.5_Fe_0.5_O_5+δ_ Electrodes,” Ceramics International 47, no. 6 (2021): 7790–7797, 10.1016/j.ceramint.2020.11.124.

[15] H. Ding , W. Wu , C. Jiang , et al., “Self‐Sustainable Protonic Ceramic Electrochemical Cells Using a Triple Conducting Electrode for Hydrogen and Power Production,” Nature Communications 11, no. 1 (2020): 1907, 10.1038/s41467-020-15677-z. PMC7171140 32312963

[16] E. Vøllestad , R. Strandbakke , M. Tarach , et al., “Mixed Proton and Electron Conducting Double Perovskite Anodes for Stable and Efficient Tubular Proton Ceramic Electrolysers,” Nature Materials 18, no. 7 (2019): 752–759, 10.1038/s41563-019-0388-2. 31160804

[17] X. Li , J. Feng , N. Sun , et al., “A‐Site High‐Entropy Engineering of Oxygen Electrode: A Promising Route to Durable and Active Reversible Solid Oxide Cells,” Advanced Materials 38 (2026): 21863, 10.1002/adma.202521863. 41622901

[18] F. Li , L. Jiang , R. Zeng , F. Wang , Y. Xu , and Y. Huang , “Hetero‐Structured La_0.5_Sr_0.5_CoO_3–δ_–/LaSrCoO_4±δ_ Cathode With High Electro‐Catalytic Activity for Solid‐Oxide Fuel Cells,” International Journal of Hydrogen Energy 42, no. 49 (2017): 29463–29471, 10.1016/j.ijhydene.2017.10.001.

[19] Y. Xu , K. Xu , F. Zhu , et al., “A Low‐Lewis‐Acid‐Strength Cation Cs^+^‐Doped Double Perovskite for Fast and Durable Oxygen Reduction/Evolutions on Protonic Ceramic Cells,” ACS Energy Letters 8, no. 10 (2023): 4145–4155, 10.1021/acsenergylett.3c01722.

[20] Y. Zhang , X. Hao , J. Liu , et al., “Effectively Boosting Hydration Capacity and Oxygen Reduction Activity of Cobalt‐free Perovskite Cathode by K^+^ Doping Strategy for Protonic Ceramic Fuel Cells,” Ceramics International 50, no. 3 (2024): 4746–4755, 10.1016/j.ceramint.2023.11.219.

[21] C. Oses , C. Toher , and S. Curtarolo , “High‐Entropy Ceramics,” Nature Reviews Materials 5, no. 4 (2020): 295–309, 10.1038/s41578-019-0170-8.

[22] D. B. Miracle and O. N. Senkov , “A Critical Review of High Entropy Alloys and Related Concepts,” Acta Materialia 122 (2017): 448–511, 10.1016/j.actamat.2016.08.081.

[23] O. F. Dippo and K. S. Vecchio , “A Universal Configurational Entropy Metric for High‐Entropy Materials,” Scripta Materialia 201 (2021): 113974, 10.1016/j.scriptamat.2021.113974.

[24] Y. Yang , H. Bao , H. Ni , et al., “A Novel Facile Strategy to Suppress Sr Segregation for High‐Entropy Stabilized La_0·8_Sr_0·2_MnO_3‐δ_ Cathode,” Journal of Power Sources 482 (2021): 228959, 10.1016/j.jpowsour.2020.228959.

[25] S. H. Albedwawi , A. AlJaberi , G. N. Haidemenopoulos , and K. Polychronopoulou , “High Entropy Oxides‐Exploring a Paradigm of Promising Catalysts: A Review,” Materials & Design 202 (2021): 109534, 10.1016/j.matdes.2021.109534.

[26] J. Dąbrowa , A. Olszewska , A. Falkenstein , et al., “An Innovative Approach to Design SOFC Air Electrode Materials: High Entropy La_1−x_Srx(Co,Cr,Fe,Mn,Ni)O_3−δ_ (x = 0, 0.1, 0.2, 0.3) Perovskites Synthesized by the Sol‐Gel Method,” Journal of Materials Chemistry A 8, no. 46 (2020): 24455–24468, 10.1039/D0TA06356H.

[27] X. Han , Y. Yang , Y. Fan , et al., “New Approach to Enhance Sr‐Free Cathode Performance by High‐Entropy Multi‐Component Transition Metal Coupling,” Ceramics International 47, no. 12 (2021): 17383–17390, 10.1016/j.ceramint.2021.03.052.

[28] Z. Liu , Z. Tang , Y. Song , et al., “High‐Entropy Perovskite Oxide: A New Opportunity for Developing Highly Active and Durable Air Electrode for Reversible Protonic Ceramic Electrochemical Cells,” Nano‐Micro Letters 14, no. 1 (2022): 217, 10.1007/s40820-022-00967-6. 36352041 PMC9646682

[29] F. He , F. Zhu , D. Liu , et al., “A Reversible Perovskite Air Electrode for Active and Durable Oxygen Reduction and Evolution Reactions via the A‐Site Entropy Engineering,” Materials Today 63 (2023): 89–98, 10.1016/j.mattod.2023.02.006.

[30] F. He , Y. Zhou , T. Hu , et al., “An Efficient High‐Entropy Perovskite‐Type Air Electrode for Reversible Oxygen Reduction and Water Splitting in Protonic Ceramic Cells,” Advanced Materials 35, no. 16 (2023): 2209469, 10.1002/adma.202209469. 36722205

[31] D. Shang , B. Zhang , L. Zhang , et al., “Improving Electrocatalytic Activity Through Multi‐element Doping to A‐Site of Fe‐Based Perovskite Cathode for Solid Oxide Fuel Cells,” Chemical Engineering Journal 506 (2025): 160067, 10.1016/j.cej.2025.160067.

[32] X. Han , Y. Ling , Y. Yang , et al., “Utilizing High Entropy Effects for Developing Chromium‐Tolerance Cobalt‐Free Cathode for Solid Oxide Fuel Cells,” Advanced Functional Materials 33, no. 43 (2023): 2304728, 10.1002/adfm.202304728.

[33] Y. Zhang , B. Niu , X. Hao , et al., “Layered Oxygen‐Deficient Double Perovskite GdBaFe_2_O_5+δ_ as Electrode Material for Symmetrical Solid‐Oxide Fuel Cells,” Electrochimica Acta 370 (2021): 137807, 10.1016/j.electacta.2021.137807.

[34] H. Ding and X. Xue , “Cobalt‐Free Layered Perovskite GdBaFe_2_O_5+x_ as a Novel Cathode for Intermediate Temperature Solid Oxide Fuel Cells,” Journal of Power Sources 195, no. 15 (2010): 4718–4721, 10.1016/j.jpowsour.2010.02.027.

[35] J. Yang , Y. Lv , X. Xu , et al., “Remarkably High Proton Conductivity in Cubic Perovskite‐Related Ba_3_WO_6_ ,” Journal of Materials Chemistry A 10, no. 31 (2022): 16697–16703, 10.1039/D2TA04282G.

[36] S. Fop , K. S. McCombie , E. J. Wildman , et al., “High Oxide Ion and Proton Conductivity in a Disordered Hexagonal Perovskite,” Nature Materials 19, no. 7 (2020): 752–757, 10.1038/s41563-020-0629-4. 32123332

[37] M. Yashima , “Crystal Structures, Structural Disorders and Diffusion Paths of Ionic Conductors From Diffraction Experiments,” Solid State Ionics 179, no. 21 (2008): 797–803, 10.1016/j.ssi.2007.12.099.

[38] M. Liang , Y. Zhu , Y. Song , et al., “A New Durable Surface Nanoparticles‐Modified Perovskite Cathode for Protonic Ceramic Fuel Cells From Selective Cation Exsolution Under Oxidizing Atmosphere,” Advanced Materials 34, no. 10 (2022): 2106379, 10.1002/adma.202106379. 34962667

[39] K. Lankauf , A. Mroziński , P. Błaszczak , et al., “The Effect of Fe on Chemical Stability and Oxygen Evolution Performance of High Surface Area SrTi_x‐1_Fe_x_O_3‐δ_ Mixed Ionic‐Electronic Conductors in Alkaline media,” International Journal of Hydrogen Energy 46, no. 56 (2021): 28575–28590, 10.1016/j.ijhydene.2021.06.088.

[40] J. Yu , Q. Liu , S. Wang , et al., “Spin‐State Tuning in PrFeO_3‐δ_ Perovskite for High‐Temperature Oxygen Evolution Reaction,” Journal of the American Chemical Society 147, no. 36 (2025): 33086–33096, 10.1021/jacs.5c10937. 40859643

[41] F. Zhu , Z. Du , K. Xu , et al., “Entropy and Composition Regulations of Air Electrodes Enable Efficient Oxygen Reduction and Evolution Reactions for Reversible Solid Oxide Cells,” Advanced Energy Materials 14, no. 37 (2024): 2401048, 10.1002/aenm.202401048.

[42] Y.‐L. Huang , A. M. Hussain , and E. D. Wachsman , “Nanoscale Cathode Modification for High Performance and Stable Low‐Temperature Solid Oxide Fuel Cells (SOFCs),” Nano Energy 49 (2018): 186–192, 10.1016/j.nanoen.2018.04.028.

[43] X. Han , K. Li , Q. Shao , et al., “A Durable and Highly Active Oxygen Electrode for Solid Oxide Cells: New Insight Into Segregation Suppression of Layered Perovskite,” Advanced Materials 37, no. 28 (2025): 2502068, 10.1002/adma.202502068. 40318093

[44] X. Chen , N. Yu , Y. Song , et al., “Synergistic Bulk and Surface Engineering for Expeditious and Durable Reversible Protonic Ceramic Electrochemical Cells Air Electrode,” Advanced Materials 36, no. 32 (2024): 2403998, 10.1002/adma.202403998. 38801699

[45] F. Zhu , K. Xu , F. He , et al., “An Active and Contaminants‐Tolerant High‐Entropy Electrode for Ceramic Fuel Cells,” ACS Energy Letters 9, no. 2 (2024): 556–567, 10.1021/acsenergylett.4c00037.

[46] Z. Liu , H. Xie , Y. Zhang , et al., “Towards High Performance Durable Ceramic Fuel Cells Using a Triple Conducting Perovskite Cathode,” Applied Catalysis B: Environment and Energy 346 (2024): 123678, 10.1016/j.apcatb.2023.123678.

[47] K. Liu , F. Lu , X. Jia , H. He , J. Su , and B. Cai , “A High Performance Thermal Expansion Offset Composite Cathode for IT‐SOFCs,” Journal of Materials Chemistry A 10, no. 45 (2022): 24410–24421, 10.1039/D2TA04899J.

[48] B. Admasu Beshiwork , X. Wan , M. Xu , et al., “A Defective Iron‐Based Perovskite Cathode for High‐Performance IT‐SOFCs: Tailoring the Oxygen Vacancies Using Nb/Ta Co‐Doping,” Journal of Energy Chemistry 88 (2024): 306–316, 10.1016/j.jechem.2023.09.015.

[49] K. Pei , Y. Zhou , K. Xu , et al., “Enhanced Cr‐Tolerance of an SOFC Cathode by an Efficient Electro‐Catalyst Coating,” Nano Energy 72 (2020): 104704, 10.1016/j.nanoen.2020.104704.

[50] Y. Li , N. Mushtaq , Y. Chen , et al., “Revisiting Mo‐Doped SrFeO_3‐δ_ Perovskite: The Origination of Cathodic Activity and Longevity for Intermediate‐Temperature Solid Oxide Fuel Cells,” Advanced Functional Materials 35, no. 3 (2025): 2411025, 10.1002/adfm.202411025.

[51] Z. Xia , Y. Zhang , X. Xiong , et al., “Realizing B‐Site High‐Entropy Air Electrode for Superior Reversible Solid Oxide Cells,” Applied Catalysis B: Environment and Energy 357 (2024): 124314, 10.1016/j.apcatb.2024.124314.

[52] C. Duan , J. Tong , M. Shang , et al., “Readily Processed Protonic Ceramic Fuel Cells With High Performance at Low Temperatures,” Science 349, no. 6254 (2015): 1321–1326, 10.1126/science.aab3987. 26217064

[53] S. Zhao , W. Ma , W. Wang , et al., “Reverse Atom Capture on Perovskite Surface Enabling Robust and Efficient Cathode for Protonic Ceramic Fuel Cells,” Advanced Materials 36, no. 27 (2024): 2405052, 10.1002/adma.202405052. 38652767

[54] H. Zhang , K. Xu , F. He , et al., “Surface Regulating of a Double‐Perovskite Electrode for Protonic Ceramic Fuel Cells to Enhance Oxygen Reduction Activity and Contaminants Poisoning Tolerance,” Advanced Energy Materials 12, no. 26 (2022): 2200761, 10.1002/aenm.202200761.

[55] D. Zou , Y. Yi , Y. Song , et al., “The BaCe_0.16_Y_0.04_Fe_0.8_O_3−δ_ Nanocomposite: A new High‐Performance Cobalt‐Free Triple‐Conducting Cathode for Protonic Ceramic Fuel Cells Operating at Reduced Temperatures,” Journal of Materials Chemistry A 10, no. 10 (2022): 5381–5390, 10.1039/D1TA10652J.

[56] G.‐M. Park , K. Park , M. Jo , et al., “Nickel‐Doped NdBa_0.5_Sr_0.5_Co_1.5_Fe_0.5_O_5+δ_ Oxygen Electrode Material for High Performance Reversible Protonic Ceramic Cells,” Journal of Alloys and Compounds 968 (2023): 171987, 10.1016/j.jallcom.2023.171987.

[57] J. Wang , Z. Li , H. Zang , et al., “BaZr_0.1_Fe_0.9_‐xNixO_3‐δ_ Cubic Perovskite Oxides for Protonic Ceramic Fuel Cell Cathodes,” International Journal of Hydrogen Energy 47, no. 15 (2022): 9395–9407, 10.1016/j.ijhydene.2022.01.012.

[58] J. Li , X. Zhou , C. Wu , et al., “Self‐Stabilized Hybrid Cathode for Solid Oxide Fuel Cell: A‐Site Deficient Perovskite Coating as Solid Solution for Strontium Diffusion,” Chemical Engineering Journal 438 (2022): 135446, 10.1016/j.cej.2022.135446.

[59] J. Bai , D. Zhou , L. Niu , et al., “Preparation of High‐Performance Multiphase Heterostructures IT‐SOFC Cathode Materials by Pr‐Induced In Situ Assembly,” Applied Catalysis B: Environment and Energy 355 (2024): 124174, 10.1016/j.apcatb.2024.124174.

[60] M. Viviani , M. T. Buscaglia , P. Nanni , R. Parodi , G. Gemme , and A. Dacca , “XPS Investigation of Surface Properties of Ba(_1‐x_)Sr_x_TiO_3_ Powders Prepared by Low Temperature Aqueous Synthesis,” Journal of the European Ceramic Society 19, no. 6 (1999): 1047–1051, 10.1016/S0955-2219(98)00371-9.

[61] Y. Xu , H. Zhang , K. Xu , et al., “An Efficient Trifunctional Spinel‐Based Electrode for Oxygen Reduction/Evolution Reactions and Nonoxidative Ethane Dehydrogenation on Protonic Ceramic Electrochemical Cells,” Advanced Materials 36, no. 40 (2024): 2408044, 10.1002/adma.202408044. 39194395

