# Supplementary material for: Unraveling the Synergistic Activity‐Stability Enhancement of a High‐Entropy Perovskite Air Electrode for Dual Ceramic Electrochemical Cells
Source: Adv Sci (Weinh). 2026 Mar 31;13(34):e75100. doi: 10.1002/advs.75100 (PMC13285115; doi:10.1002/advs.75100)
Supplement: Supplementary file 1 — Supporting File: advs75100‐sup‐0001‐SuppMat.docx. [file ADVS-13-e75100-s001.docx]

Supporting Information

**Unraveling the Synergistic Activity-Stability Enhancement of a High-Entropy Perovskite Air Electrode for Dual Ceramic Electrochemical Cells**

*Ying Zhang, Yibei Wang, Zhilin Liu, Kuo Hu, Yaowen Wang, Zhen Wang, Xiyang Wang, Bingbing Niu,** *Wenquan Wang,* and Tianmin He**

Y. Zhang, Z. Wang, W. Wang, T. He

Key Laboratory of Physics and Technology for Advanced Batteries, Ministry of Education, College of Physics, Jilin University, Changchun 130012, China

E-mail: [wangwq@jlu.edu.c](mailto:wangwq@jlu.edu.cn)n; [hetm@jlu.edu.cn](mailto:hetm@jlu.edu.cn)

Y. Wang, B. Niu

School of Science, University of Science and Technology Liaoning, Anshan 114051, China

E-mail: [niubb92@foxmail.com](mailto:niubb92@foxmail.com)

Z. Liu

School of Chemistry and Chemical Engineering, Frontiers Science Center for Transformative Molecules, Shanghai Jiao Tong University, Shanghai 200240, China

K. Hu

State Key Laboratory of High Pressure and Superhard Materials, Synergetic Extreme Condition User Facility, College of Physics, Jilin University, Changchun 130012, China

Y. Wang

State Key Laboratory of Inorganic Synthesis and Preparative Chemistry, College of Chemistry, Jilin University, Changchun 130012, China

X. Wang

Department of Applied Physics, The Hong Kong Polytechnic University, Hong Kong, China

**Experimental Section**

**Materials synthesis:** The powders of GdBaFe_2_O_5+δ_ (GBF) and Gd_0.2_Pr_0.2_Ba_0.2_Sr_0.2_Ca_0.2_FeO_3−δ_ (GPBSCF) were synthesized by a sol-gel complexation method. Stoichiometric metal nitrates and ethylenediaminetetraacetic acid (EDTA) and citric acid (CA) were dissolved in deionized water with a molar ratio of total metal ions: EDTA: CA = 1:1:2. NH_3_·H_2_O was used to adjust the pH of the mixed solution to 7. The solution was heated and stirred until a gel was formed and then kept in an oven at 180 °C for 6 h. The resulting black powder was ground and heated at 600 °C for 4 h, and then the resulting powder was ground again and calcined at 1000 °C for 10 h to obtain the final sample. The synthesis process of the electrolyte powders Sm_0.2_Ce_0.8_O_1.9_ (SDC) and BaZr_0.1_Ce_0.7_Y_0.1_Yb_0.1_O_3−δ_ (BZCYYb) was similar to the above method, and the sintering temperatures of SDC and BZCYYb were 800 °C and 1100 °C, respectively, and the sintering time was 2 h.

**Cells Fabrication:** The structure of the symmetric cell exhibits electrode|SDC|electrode and electrode|BZCYYb|electrode. The SDC powders were dry pressed into 13.0 mm diameter pellets and calcined at 1400 °C for 10 h to obtain dense pellets. BZCYYb pellets were calcined at 1450 °C for 10 h to obtain dense pellets. Electrode slurry was prepared by mixing the electrode powder with terpineol (5 wt% ethyl cellulose) in a weight ratio of about 1:0.8. The electrode slurry was coated on both sides of the dense electrolyte and then sintered at 950 °C for 5 h to produce SDC-based and BZCYYb-based symmetric cells. Anode-supported single cells with structures of NiO-SDC|SDC|GPBSCF(GBF) and NiO-BZCYYb|BZCYYb|GPBSCF(GBF) were prepared by co-pressing and co-sintering anode and electrolyte powders. To make NiO-SDC anode, a mixture of NiO and SDC powders with a weight ratio of 60:40 and 10 wt% starch as a pore-forming agent was first ground in a ball mill. The anode powder was then dry pressed into a circular substrate. The SDC powder was then spread on the anode surface, co-pressed, and sintered at 1400 °C for 5 h. Finally, GPBSCF (GBF) were screen printed onto the SDC film and sintered in air at 950 °C for 5 h. The NiO-BZCYYb anode support layer was prepared by mixing NiO and BZCYYb powders in the ratio of 60:40 by weight and adding 10 wt% starch, ball-milled and dried. The densification of the co-pressed pellets was carried out at 1420 °C for 5 h. Similarly, GPBSCF (GBF) were screen printed onto the BZCYYb film (active area 0.196 cm^2^) and sintered at 950 °C in air for 5 h. In addition, silver paste and silver wire were used for current collection.

**Characterization:** The crystal structure analysis was investigated by X-ray diffraction (XRD, Rigaku Japan, SmartLab SE) using Cu Kα radiation (λ = 1.5418 Å) at room temperature (RT). Measurements were carried out over the range of 20°–80°, with a step size of 0.02° and a scanning rate of 6°/min, under operating conditions of 40 kV and 30 mA. The Rietveld refinement package GSAS was used for the structural analysis. The chemical compatibility of the GPBSCF with the SDC and BZCYYb electrolyte powders (at 1:1 weight ratio) was also investigated by XRD. In situ high-temperature X-ray diffraction (HT-XRD) measurements were performed using a X-ray diffractometer (Rigaku Japan, SmartLab SE) with a Cu Kα source. The samples were placed on a Pt strip and then heated to the appropriate temperature at a rate of 10 °C min^−1^. The diffractograms were recorded after a thermal stabilization period of 10 min at each test temperature between RT-800 °C. Microstructure of the electrode-electrolyte interface has been observed by field emission scanning electron microscopy (FESEM; Magellane-400). The crystal structure of these materials has been analysed by a high-resolution transmission electron microscopy (HRTEM; JEM-2200FS JEOL). The corresponding X-ray energy dispersive spectrum (EDS) was obtained with Bruker XFlash6T-60. The powder was first dispersed in ethanol and then floated on carbon-coated copper grids. The different elements of the prepared material were examined using X-ray photoelectron spectroscopy (XPS, ThermoFisher USA, NEXSA). The Fe L-edge and O K-edge X-ray absorption near edge structures (XANES) spectra were obtained in the total electron yield mode under vacuum superior to 5×10^−8^ Pa at the BL12B-a beamline of the National Synchrotron Radiation Laboratory (NSRL), University of Science and Technology of China, Hefei, China. The Fe K-edge and Gd L-edge XANES spectra were collected at the beamline BL14W1 of SSRF (Shanghai Synchrotron Radiation Facility) in China. The synchrotron radiation was monochromatized by a Si (111) double-crystal monochromator. To obtain the best signal-to-noise ratio, the samples diluted by LiF were pressed into 13.0 mm diameter pellets at 6 MPa. The temperature-programmed O_2_ desorption (O_2_-TPD) was carried out using a Micromeritics AutoChem II 2920 instrument. Thermogravimetric (TG) analyses were performed with a thermal analyzer (TA SDTQ-600) to study the hydration behavior of the GBF and GPBSCF in wet air. The measurement was performed with approximately 20 mg of two powders at temperatures between 30 to 950 °C with a ramp rate of 5 °C min^−1^ under a dry air atmosphere to remove surface residues. When the temperature drops to 500 °C, continue the equilibration with dry air for 80 minutes, then quickly switch to wet air to begin the hydration process. After 12 h of the hydration process, the gas was changed from wet to dry air. The gas flow has been adjusted to 50 ml min^−1^. The dilatometer (Netzsch DIL 402C) was introduced to evaluate the TEC of two materials at 30−900 °C in air with a flow rate of 60 ml min^−1^. The electrical conductivity of two samples was measured by the van der Pauw four-probe method. The Electrical conductivity relaxation (ECR) measurements were performed using a source meter (IviumStat, Ivium) at 500 to 600 °C after sudden change of oxygen partial pressure from 0.21 to 0.1 atm. The volume of the gas stream was regulated at 100 ml min^–1^. Fourier transform infrared spectroscopy (FTIR) of GPBSCF was acquired using a Bruker Vertex 80v FTIR spectrometer.

**Electrochemical measurements:** For O-SOFCs testing, the area specific resistance (ASR) values of the air electrode in the symmetrical cell were measured using the Solarton 1260 and Solarton 1287 electrochemical workstations under open circuit voltage (OCV) at a frequency ranging from 0.1 Hz to 100 kHz and signal amplitude of 10 mV at the 500–650 °C. For the SDC-based single cell, the current-voltage-power (*I*–*V*–*P*) curves were measured with an electrochemical workstation (IviumStat, Ivium) from 0 V to 1.2 V with a sequential step change of 10 mV. The power density was tested at 650–500 °C. The anode was supplied with dry H_2_ as fuel, with ambient air as the oxidant. The fuel flow rate was controlled at 30 ml min^–1^. EIS results were collected using the previously mentioned Solarton equipment with the same setting. For PCFC testing, use the same equipment to obtain data. The ASR values of the symmetrical cell were tested in a frequency range of 0.01 Hz to 100 kHz in wet air at 650–500 °C. For the BZCYYb-based single cell, the power density was tested at 650–500 °C. Wet H_2_ (3% H_2_O) was supplied to the anode as fuel, with ambient air as the oxidant. For PCEC testing, the fuel electrode was perfused with wet H_2_ (3% H_2_O) at a flow rate of 30 ml min^–1^, while the air electrode was exposed to wet air (3% H_2_O) at a flow rate of 80 ml min^–1^.

**Computational methods**

Density functional theory (DFT) calculations were performed using the Vienna ab initio Simulation Package (VASP).^[1,2]^ The exchange correlation density functional was based on the semi-local generalized gradient approximation (GGA) of Perdew–Burke–Ernzerhof (PBE).^[3]^ The projector augmented wave (PAW) potentials were used for calculation.^[4]^ Following the literature, the *U*_eff_ parameter was set to 4 eV for Fe.^[5]^ The cutoff energy for the plane wave was set to 450 eV. The Brillouin zone was sampled using Monkhorst-Pack k-point mesh automatically generated by vaspkit. The convergence criteria for self-consistent energy and atomic force were set at 10^–5^ eV and 0.02 eVÅ^–1^, respectively. Fe is contained in the material, so the spin polarization should be taken into account in the calculation. A balance was struck among the doping ratio, computational rationality, and cost for the structural optimization. Specifically, a 2×2×1 supercell was constructed for GBF, while a 5×2×1 supercell was built for GPBSCF. The substitution method was employed to incorporate gadolinium, praseodymium, barium, and calcium atoms into the cubic perovskite SrFeO_3_ structure.

**Supplementary Note 1**

The composition of the dry simulated air (hereinafter simply referred to as dry air) was 20% O_2_ and 80% N_2_. The wet air was created by passing dry air from a compressed gas bottle through a deionized water bubbler set at 20 °C (~3% H_2_O). The resulting gases have a water partial pressure (*p*_H2O_) of 0.023 *p*_0_ (*p*_0_=101.3 kPa). By supplying different proportions of pure O_2_ and N_2_, different *p*_O2_ were achieved. By changing the temperature of the deionized water bubbler, different *p*_H2O_ were achieved.


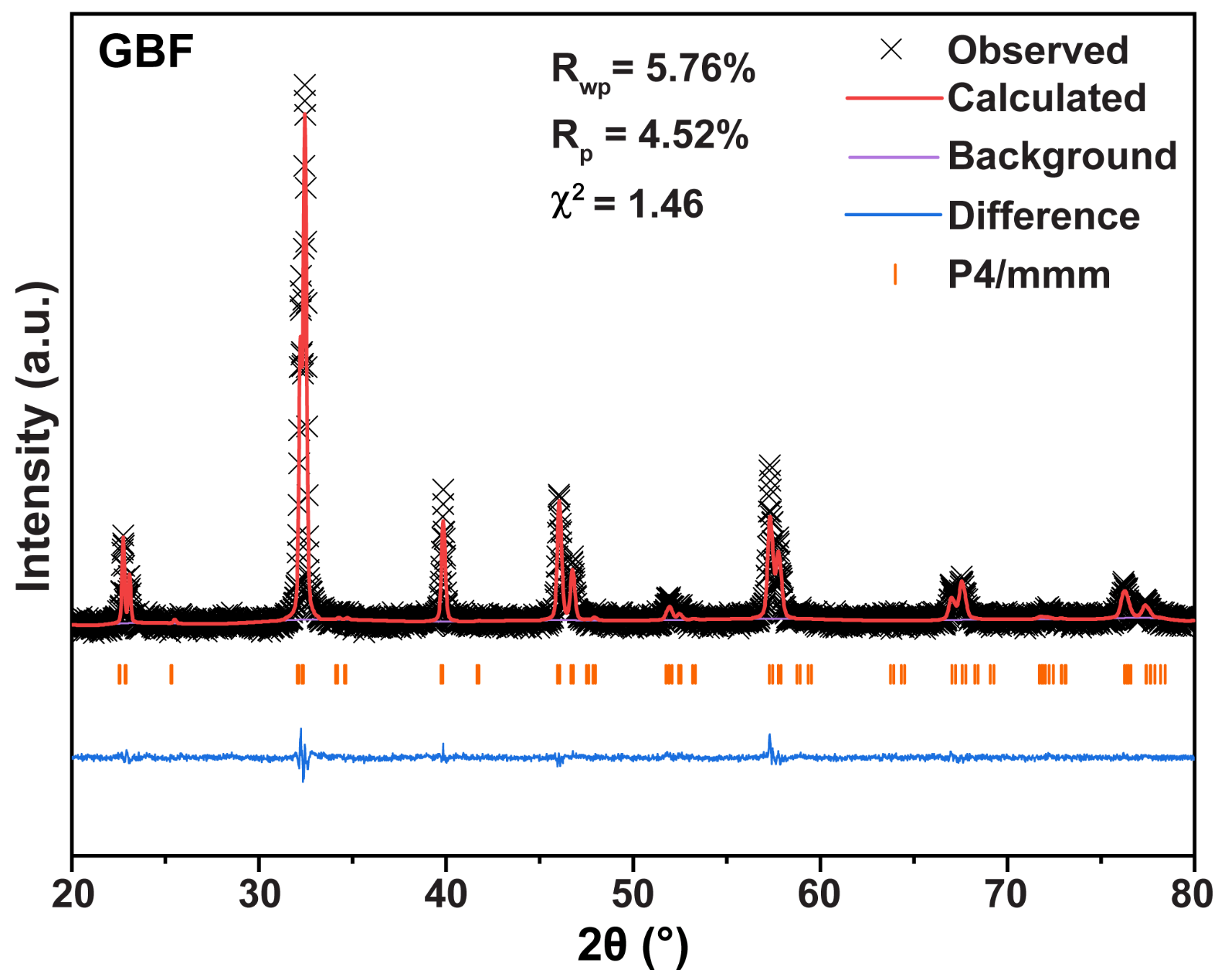


**Figure S1**. Rietveld refinement profiles of GBF sample.


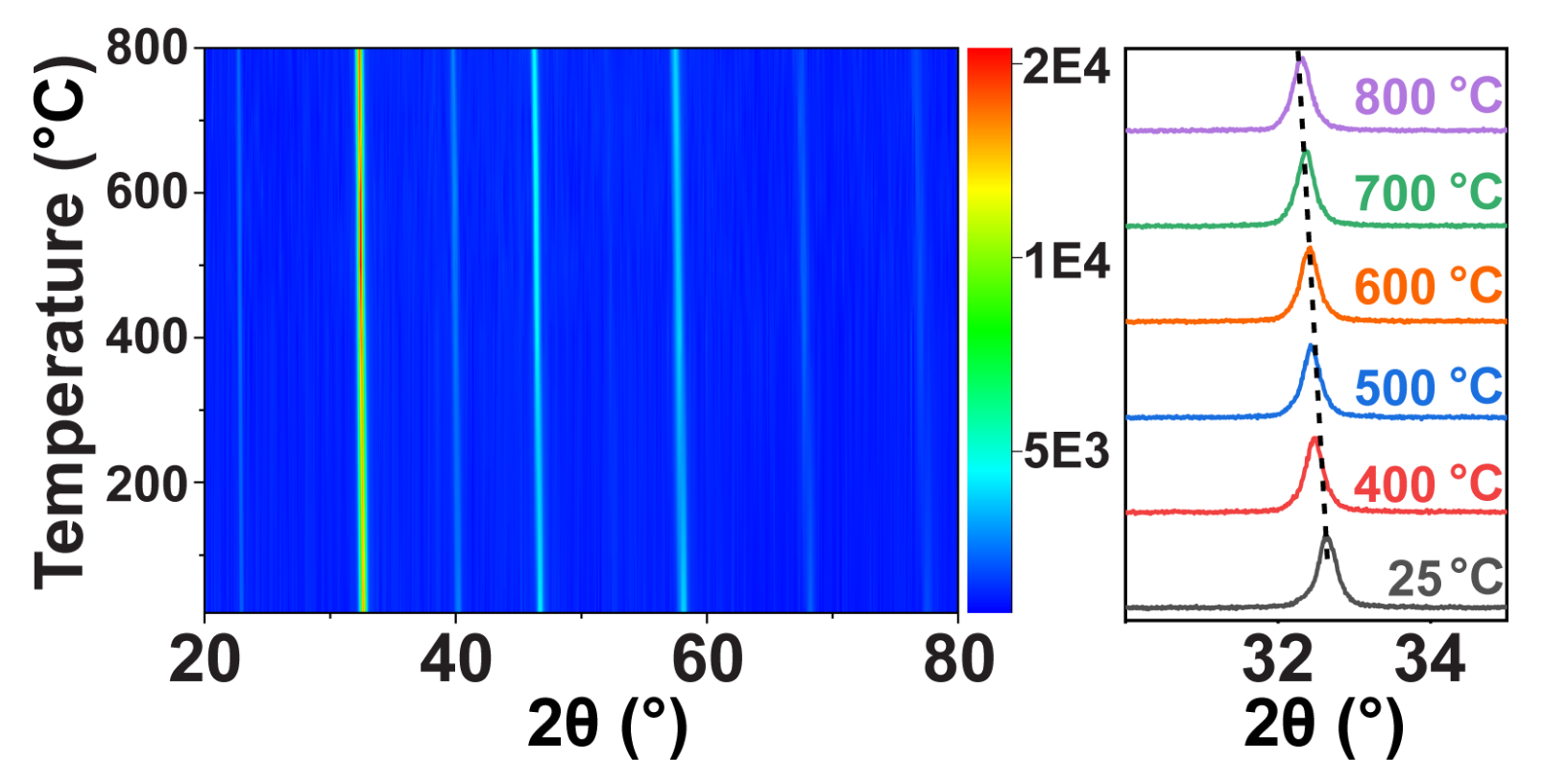


**Figure S2.** HT-XRD patterns of GPBSCF with a partial enlargement.


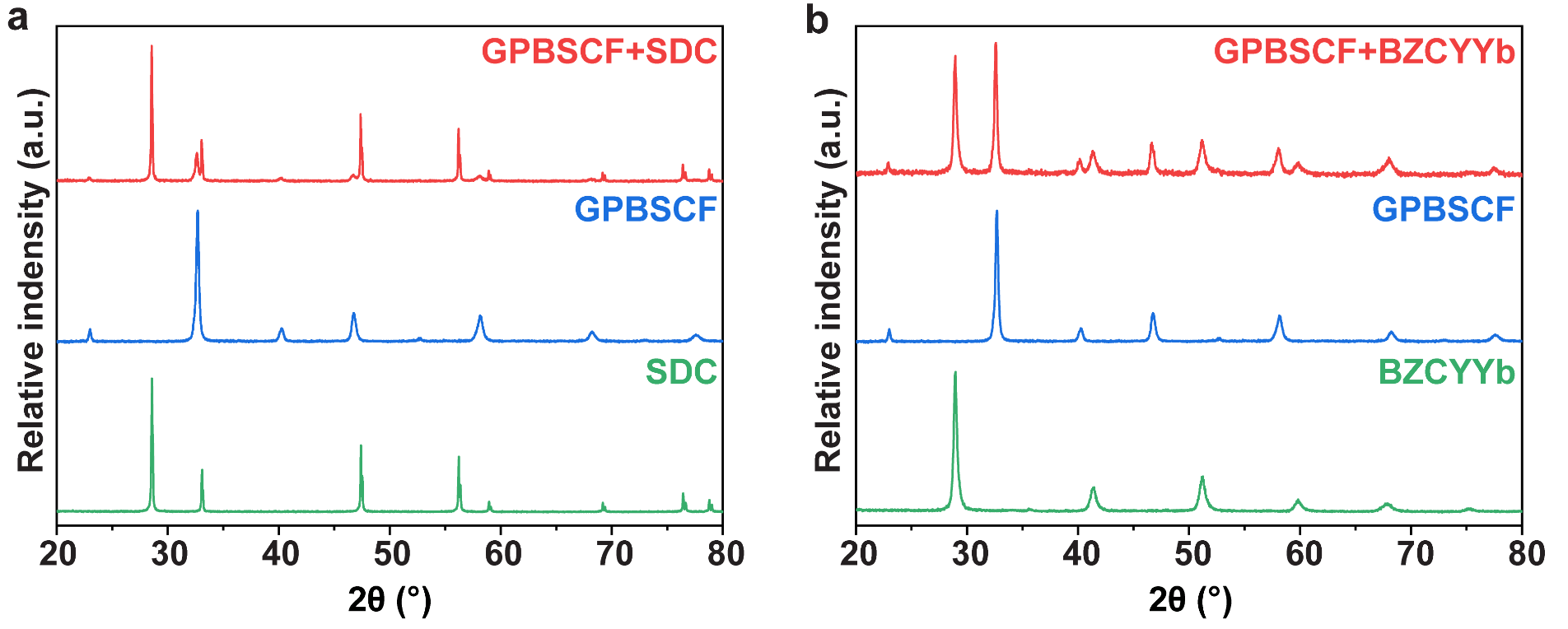


**Figure S3.** XRD patterns of a) GPBSCF electrode, SDC electrolyte and their composites and b) GPBSCF electrode, BZCYYb electrolyte and their composites after calcination at 950 °C for 5 h in air.


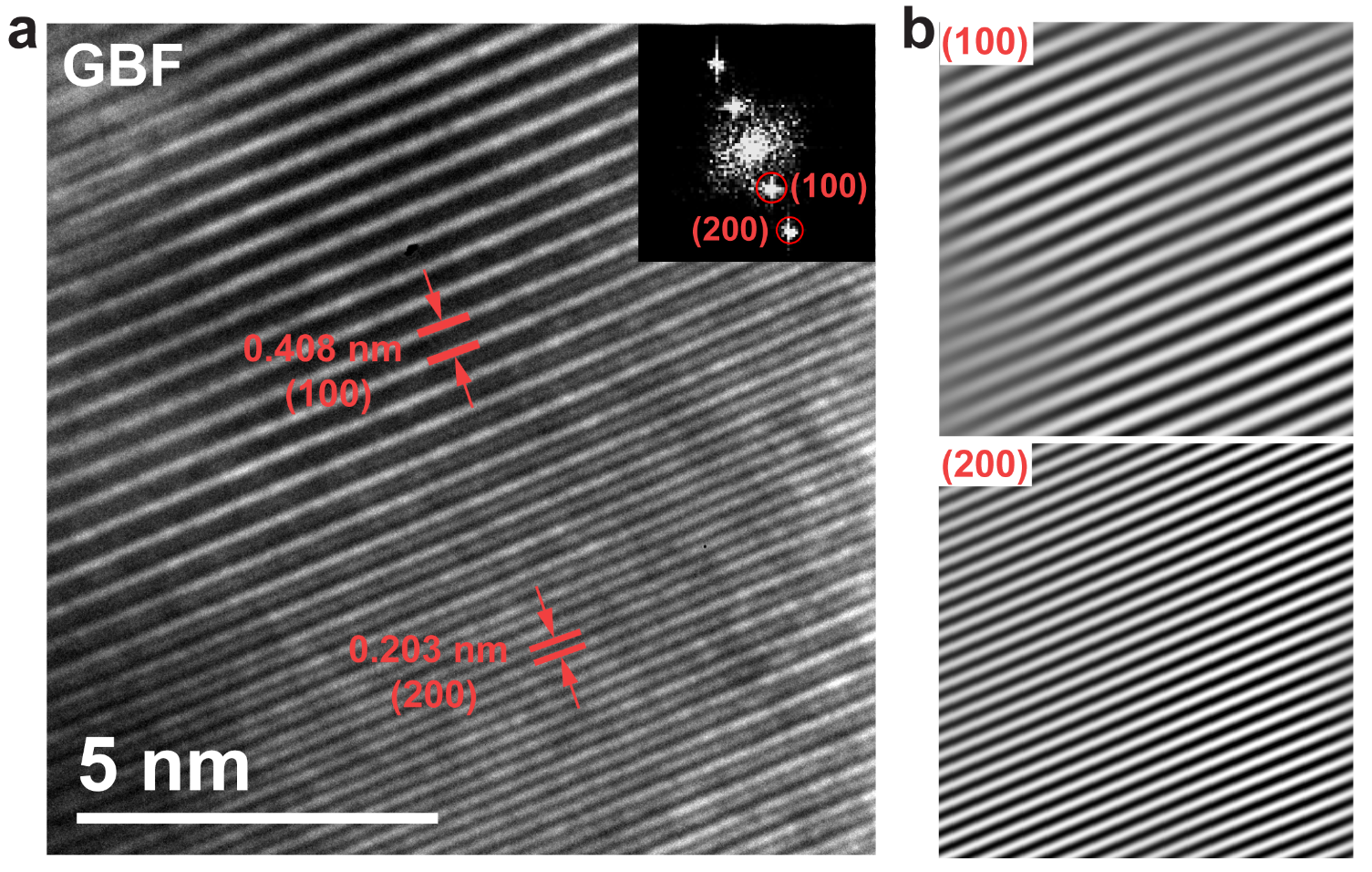


**Figure S4.** a) HR-TEM image and b) inverse fast Fourier transform filtered images of GBF sample.


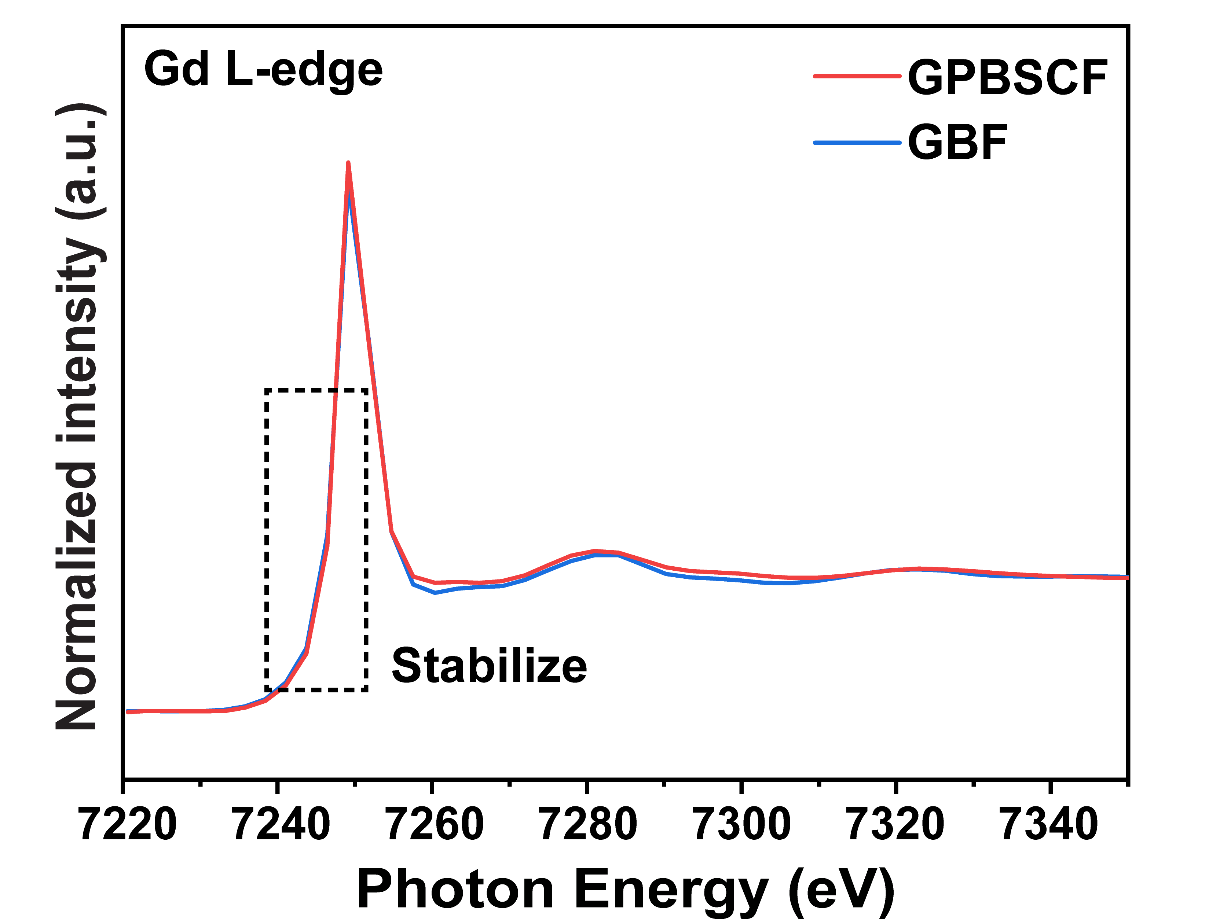


**Figure S5.** Gd L-edge X-ray absorption near edge structure of the GBF and GPBSCF samples.


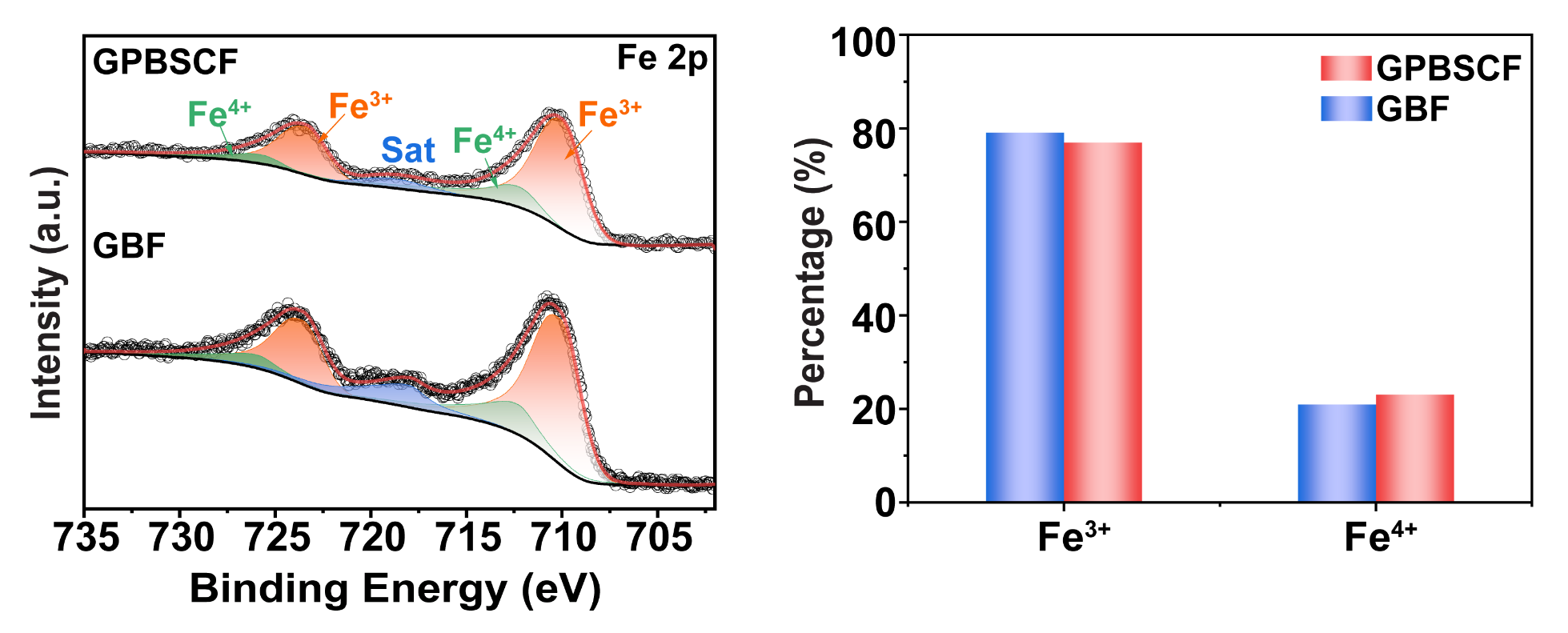


**Figure S6.** Fe 2p XPS spectra of GBF and GPBSCF samples.


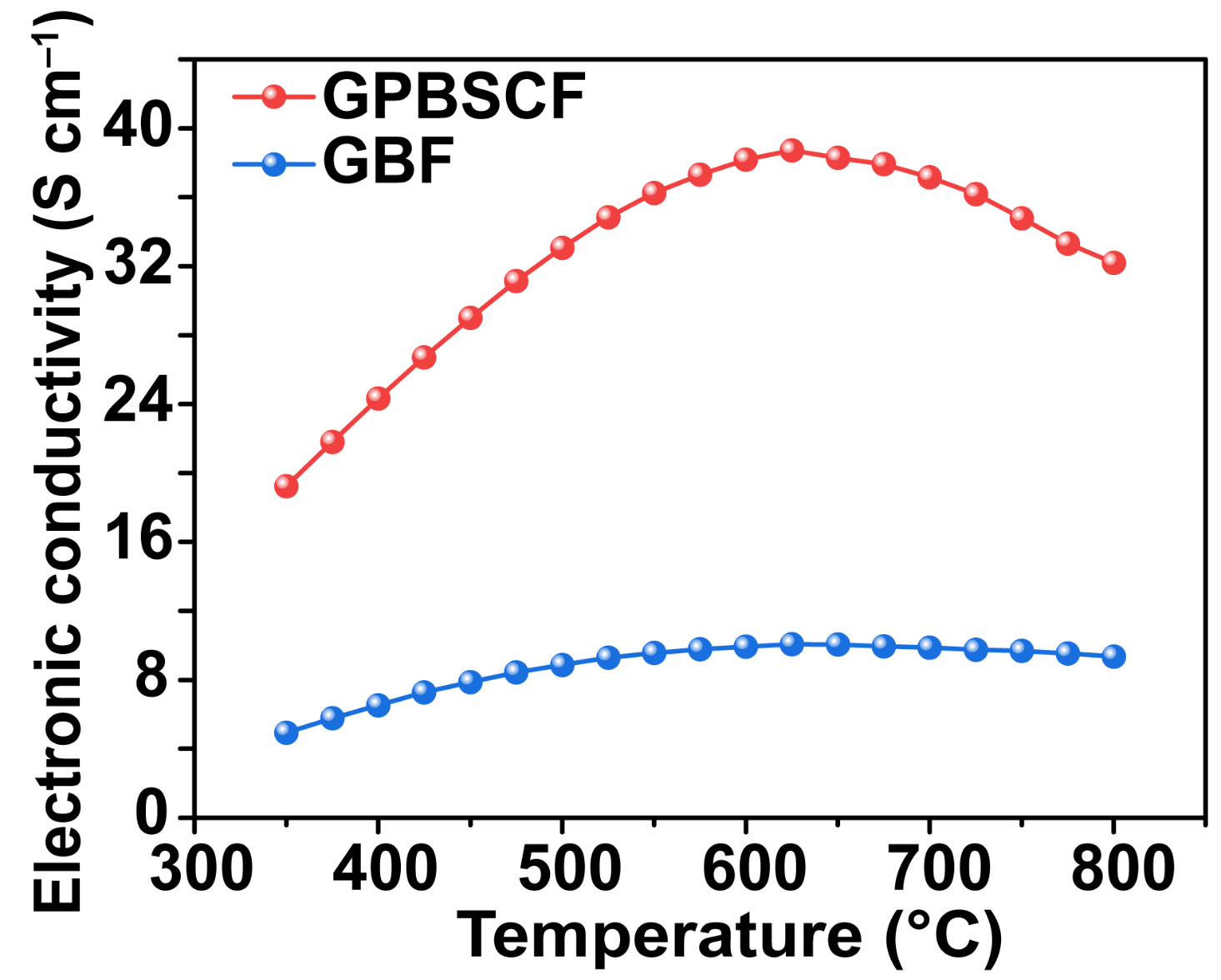


**Figure S7.** Electronic conductivity curves of GBF and GPBSCF samples.


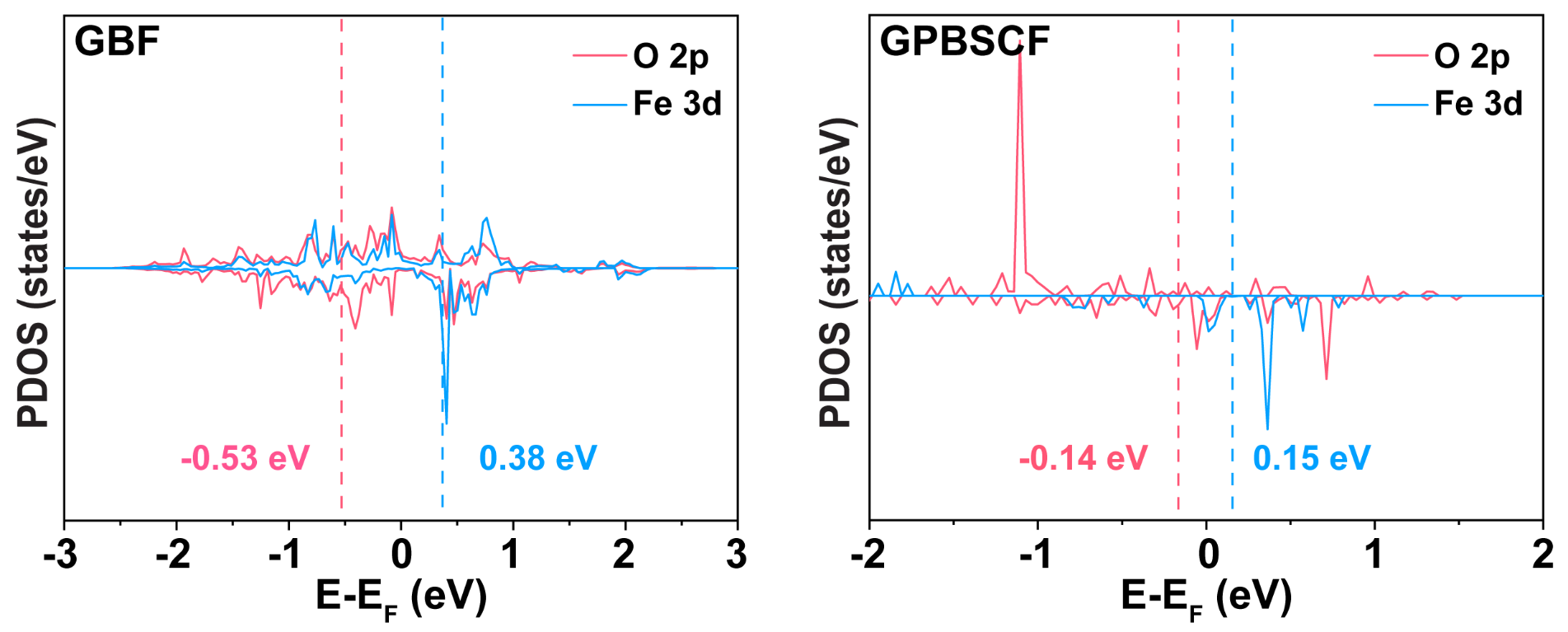


**Figure S8.** The PDOS of the O 2p and Fe 3d orbitals for GBF and GPBSCF.


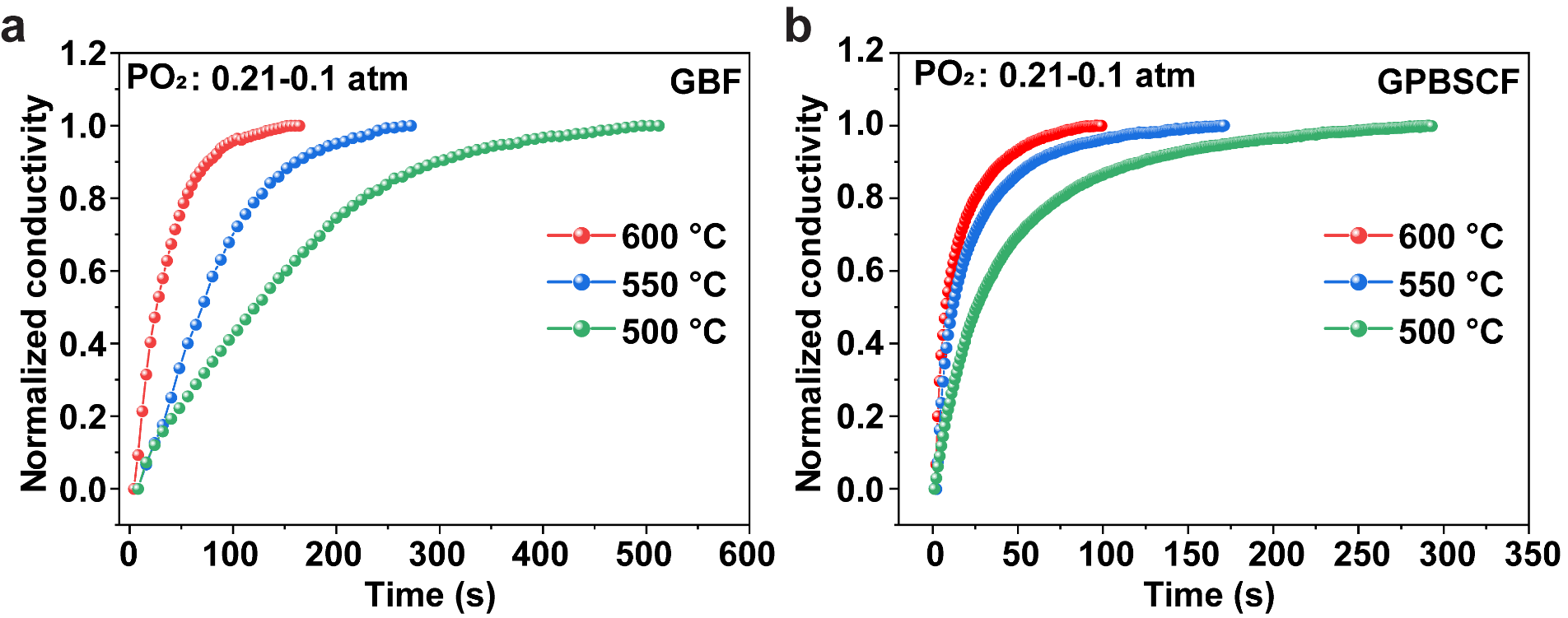


**Figure S9.** ECR curves of a) GBF and b) GPBSCF at 500-600 °C after sudden change of oxygen partial pressure from 0.21 to 0.1 atm.


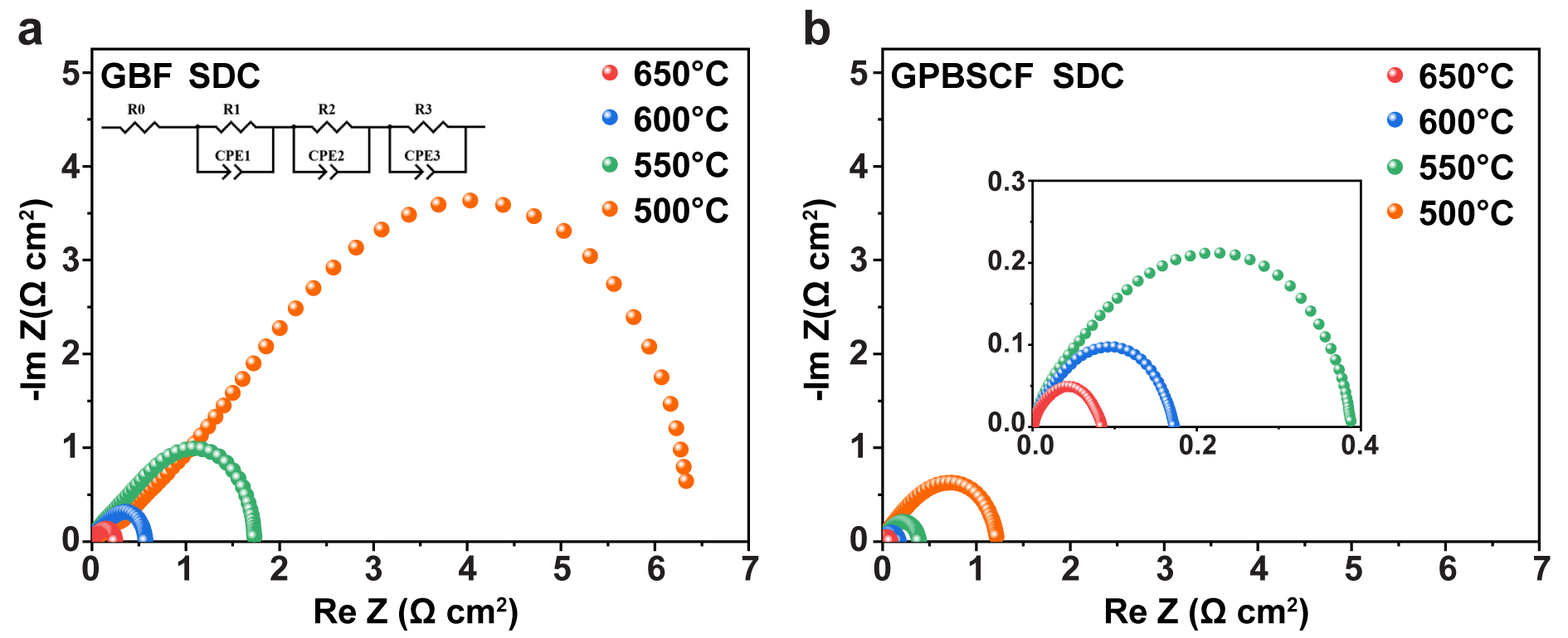


**Figure S10.** Electrochemical impedance spectra (EIS) of the SDC-based symmetrical cells with the different electrodes from 650 to 500 °C in air: a) GBF and b) GPBSCF.


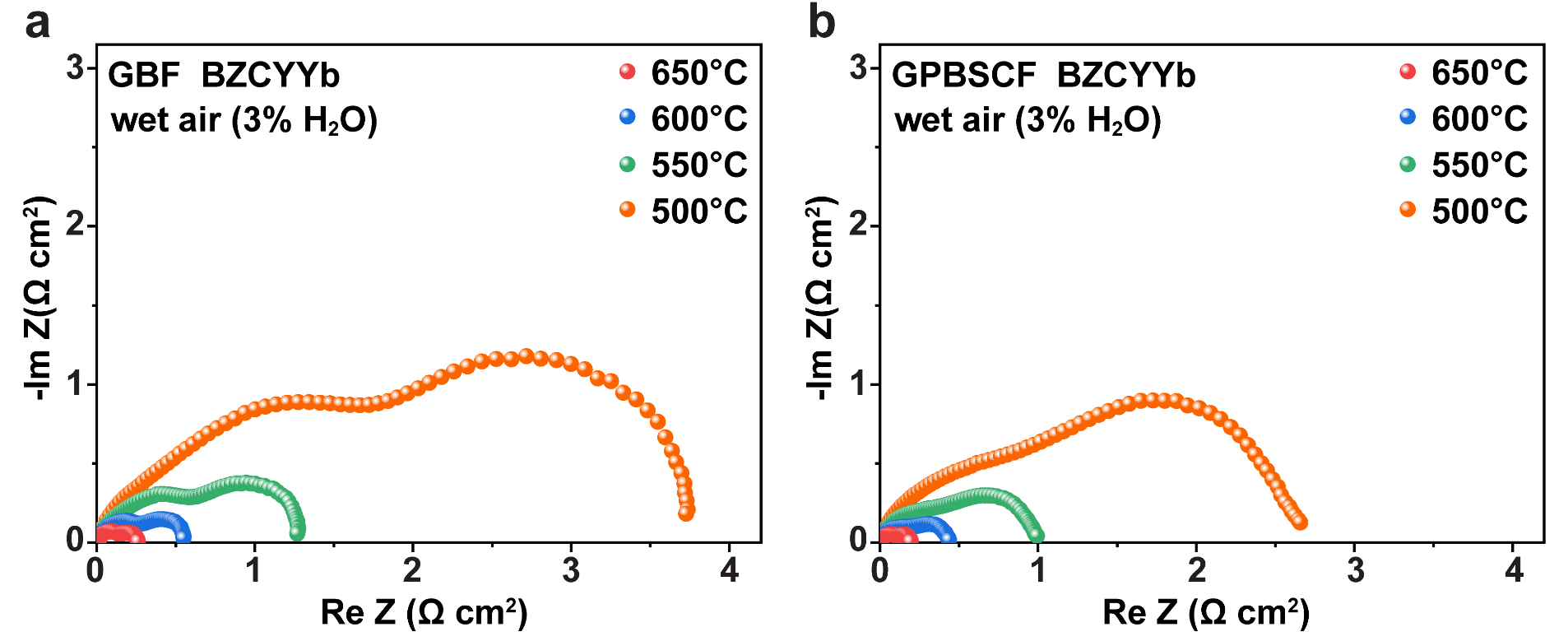


**Figure S11.** Electrochemical impedance spectra (EIS) of the BZCYYb-based symmetrical cells with the different electrodes from 650 to 500 °C in wet air: a) GBF and b) GPBSCF.


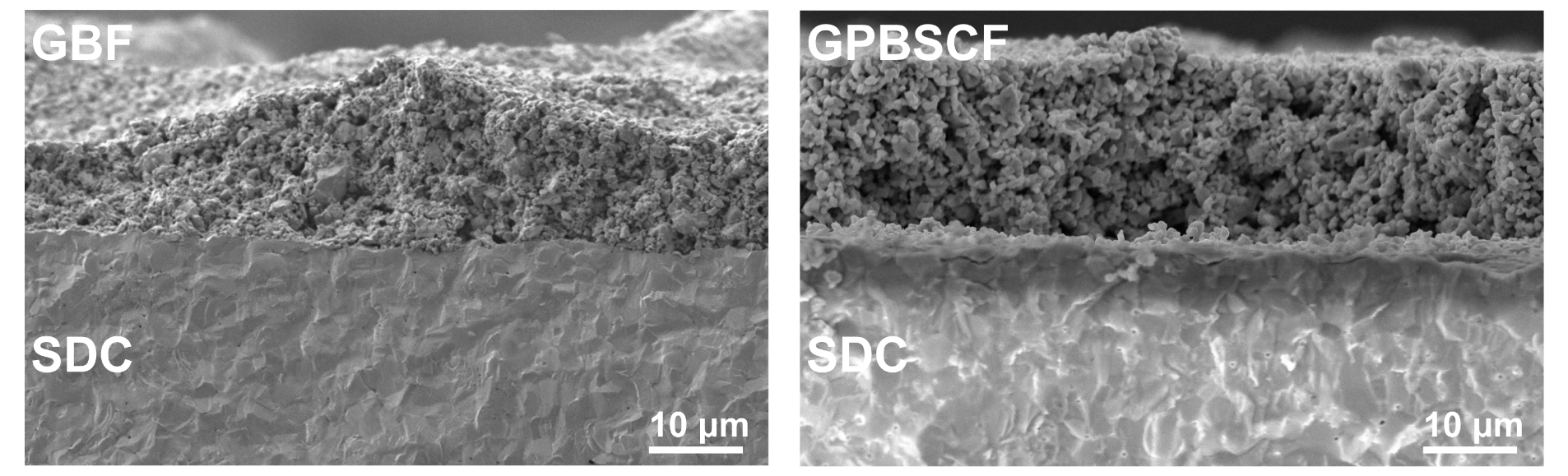


**Figure S12.** Typical cross-sectional SEM images of the SDC-based symmetric cells with GBF and GPBSCF air electrodes.


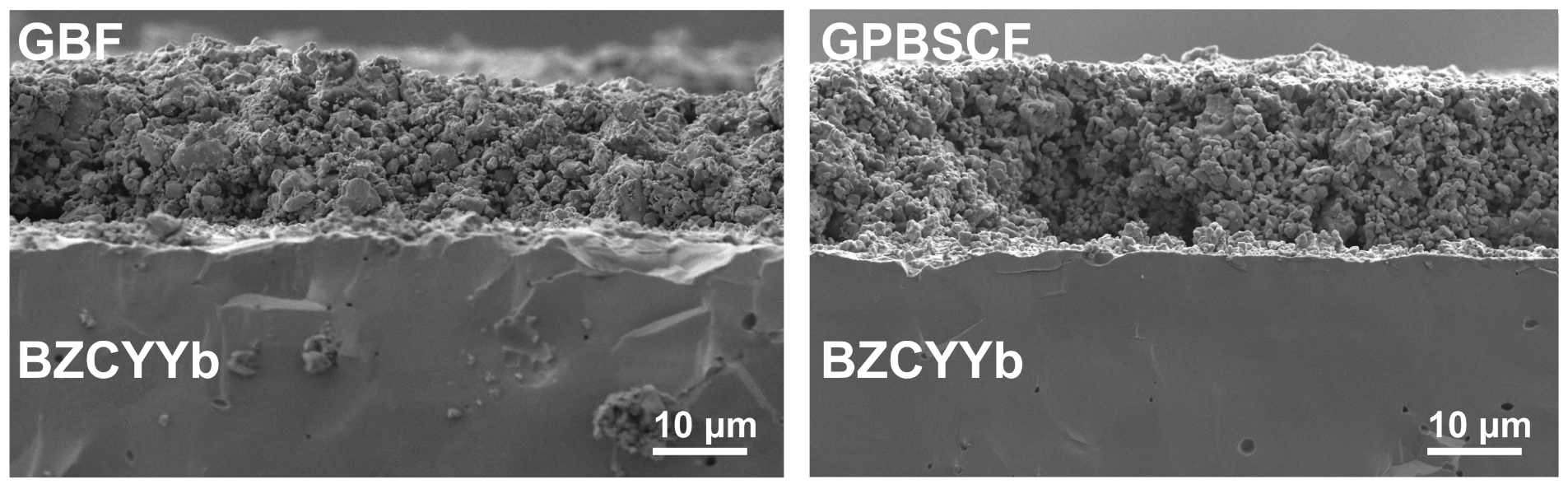


**Figure S13.** Typical cross-sectional SEM images of the BZCYYb-based symmetric cells with GBF and GPBSCF air electrodes.


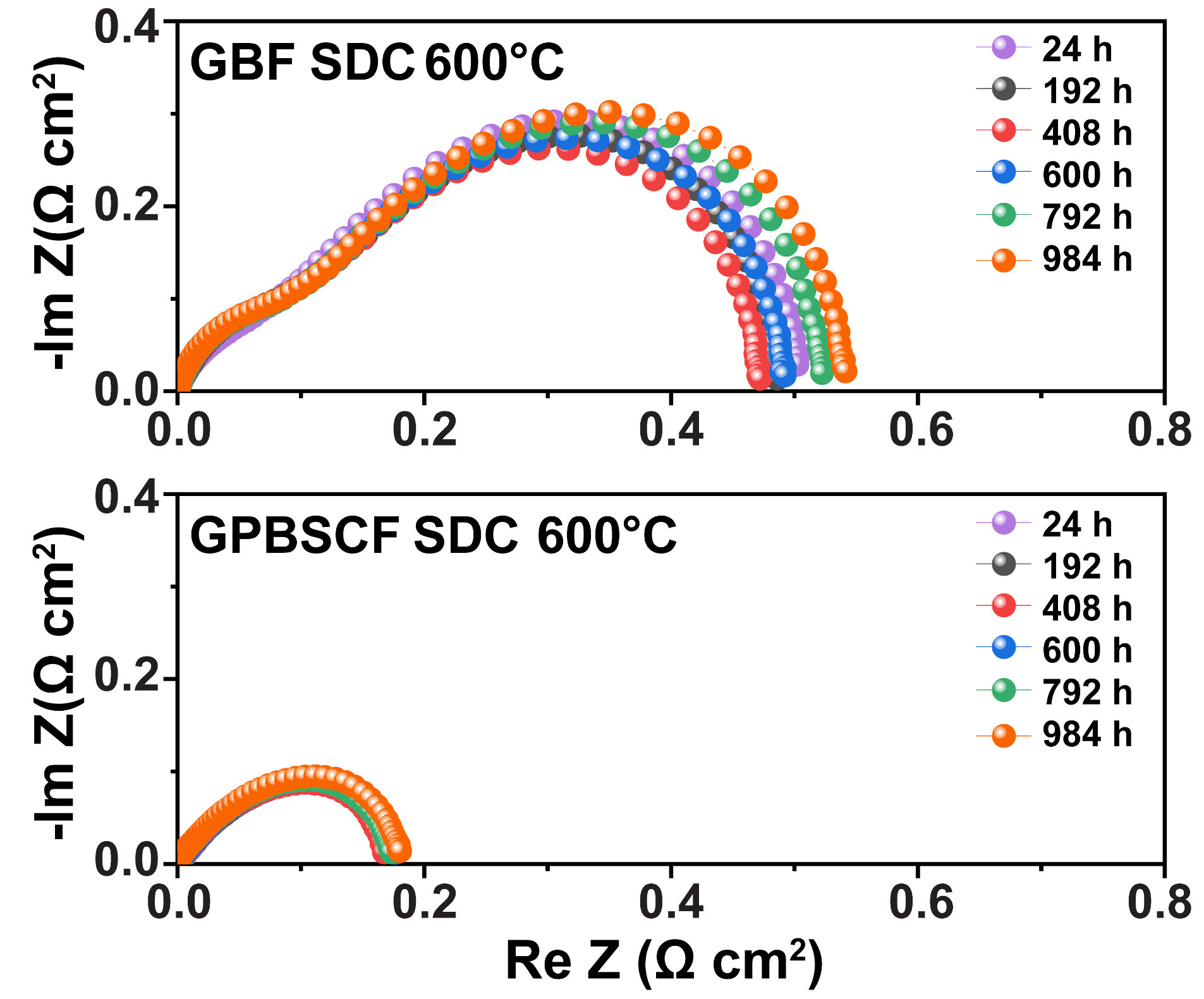


**Figure S14.** Electrochemical impedance spectra (EIS) of the SDC-based symmetrical cells with the GBF and GPBSCF electrodes at 600 °C in air.


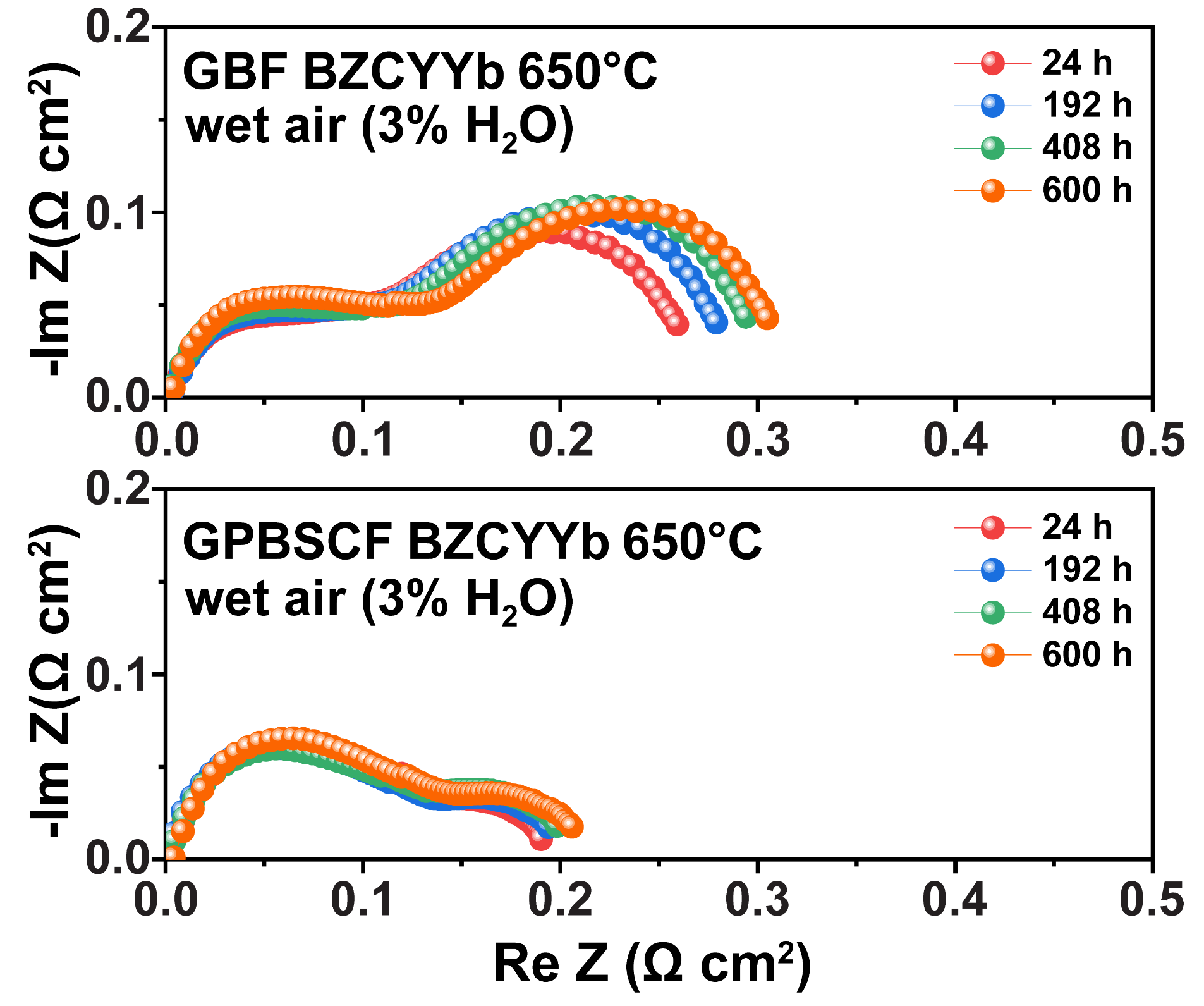


**Figure S15.** Electrochemical impedance spectra (EIS) of the BZCYYb-based symmetrical cells with the GBF and GPBSCF electrodes at 650 °C in wet air.


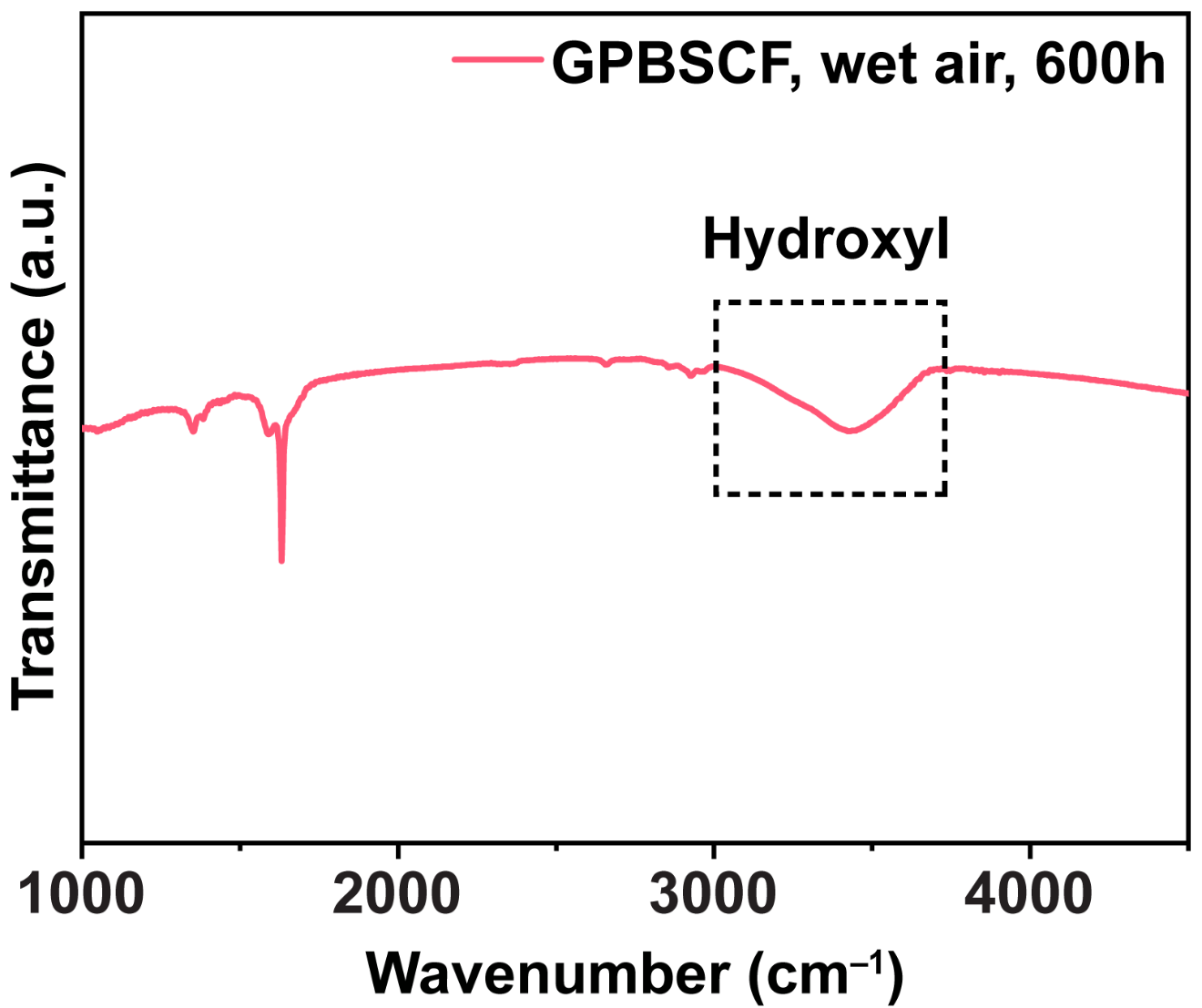


**Figure S16.** FTIR profile of GPBSCF after treatment in wet air at 650 °C for 600 h.


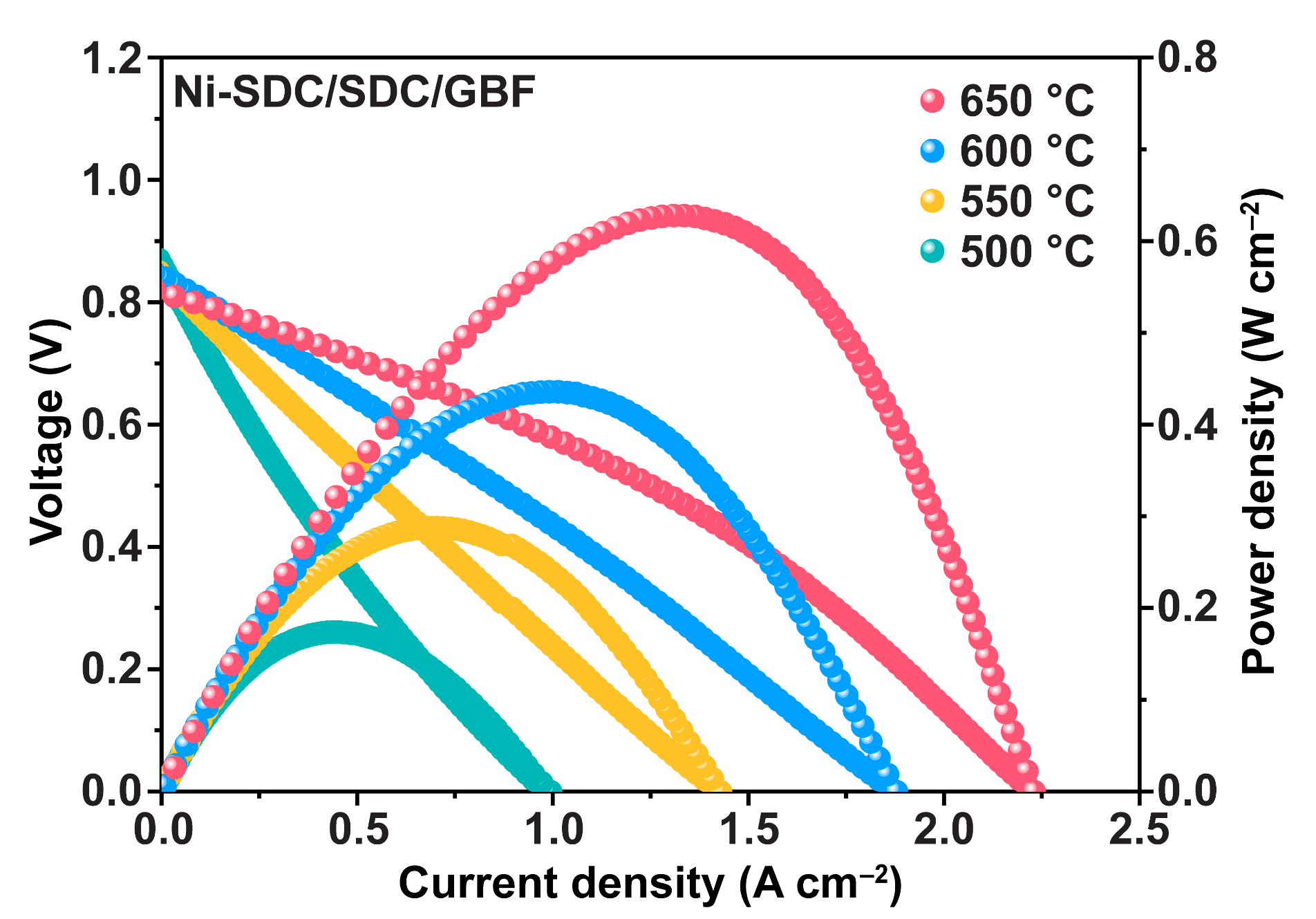


**Figure S17.** Typical *I-V-P* curves of the SDC-based single cell with GBF air electrode measured at 500−650 °C.


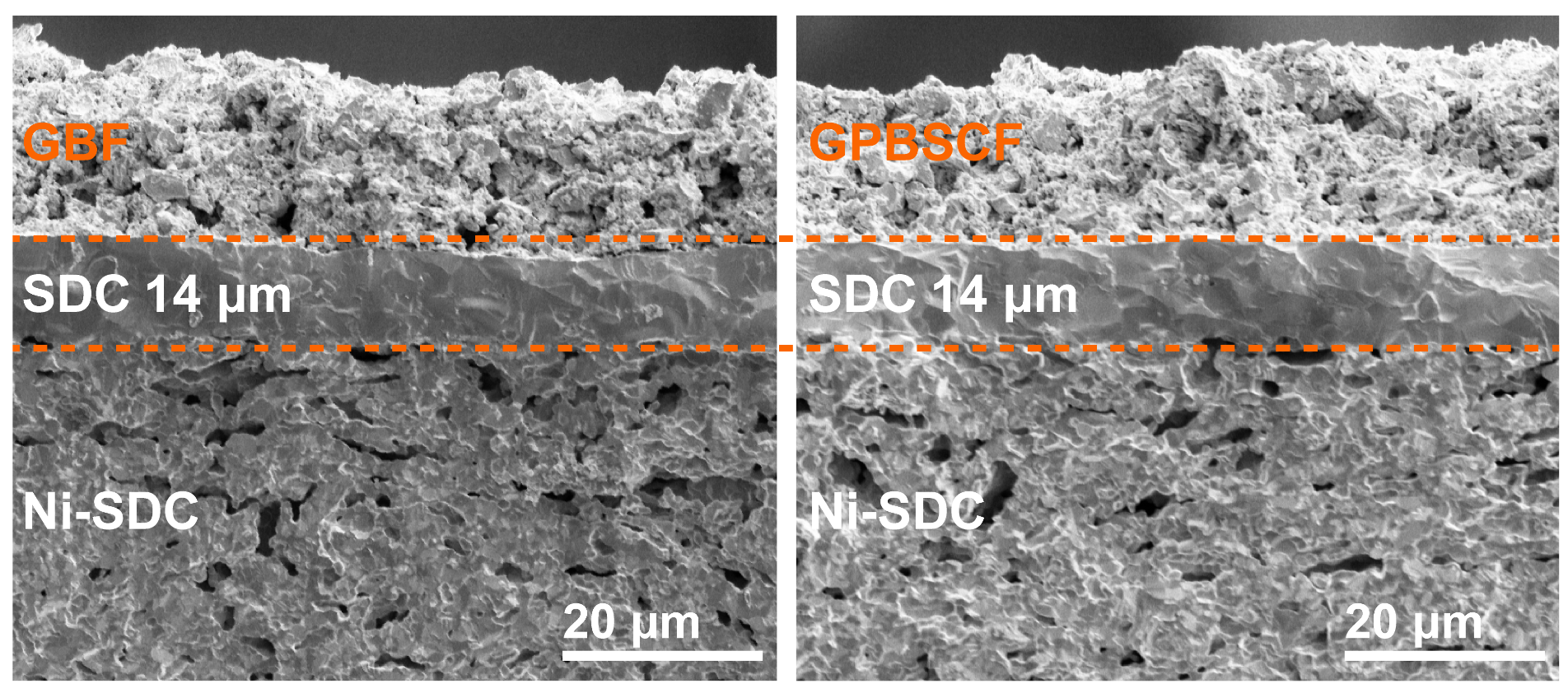


**Figure S18.** Typical cross-sectional SEM images of the Ni-SDC electrode-supported single cells with GBF and GPBSCF air electrodes.


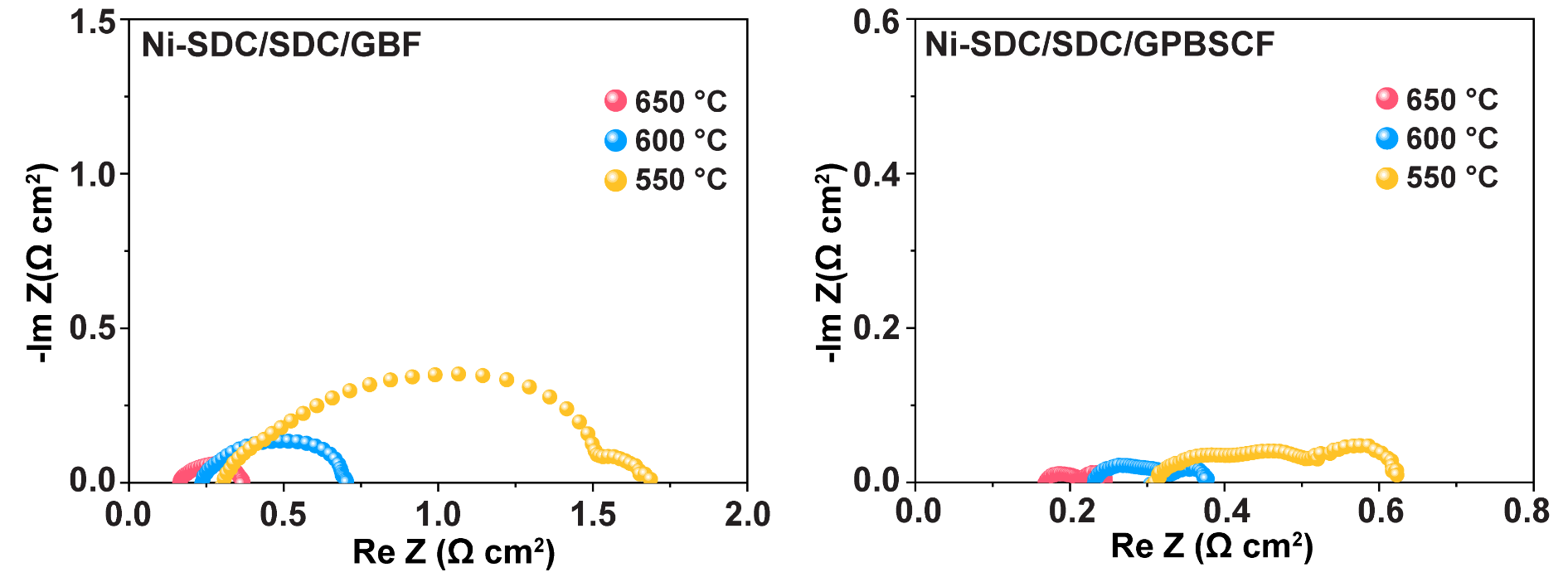


**Figure S19.** Electrochemical impedance spectra (EIS) of the SDC-based single cells with GBF and GPBSCF air electrodes measured at 550−650 °C.


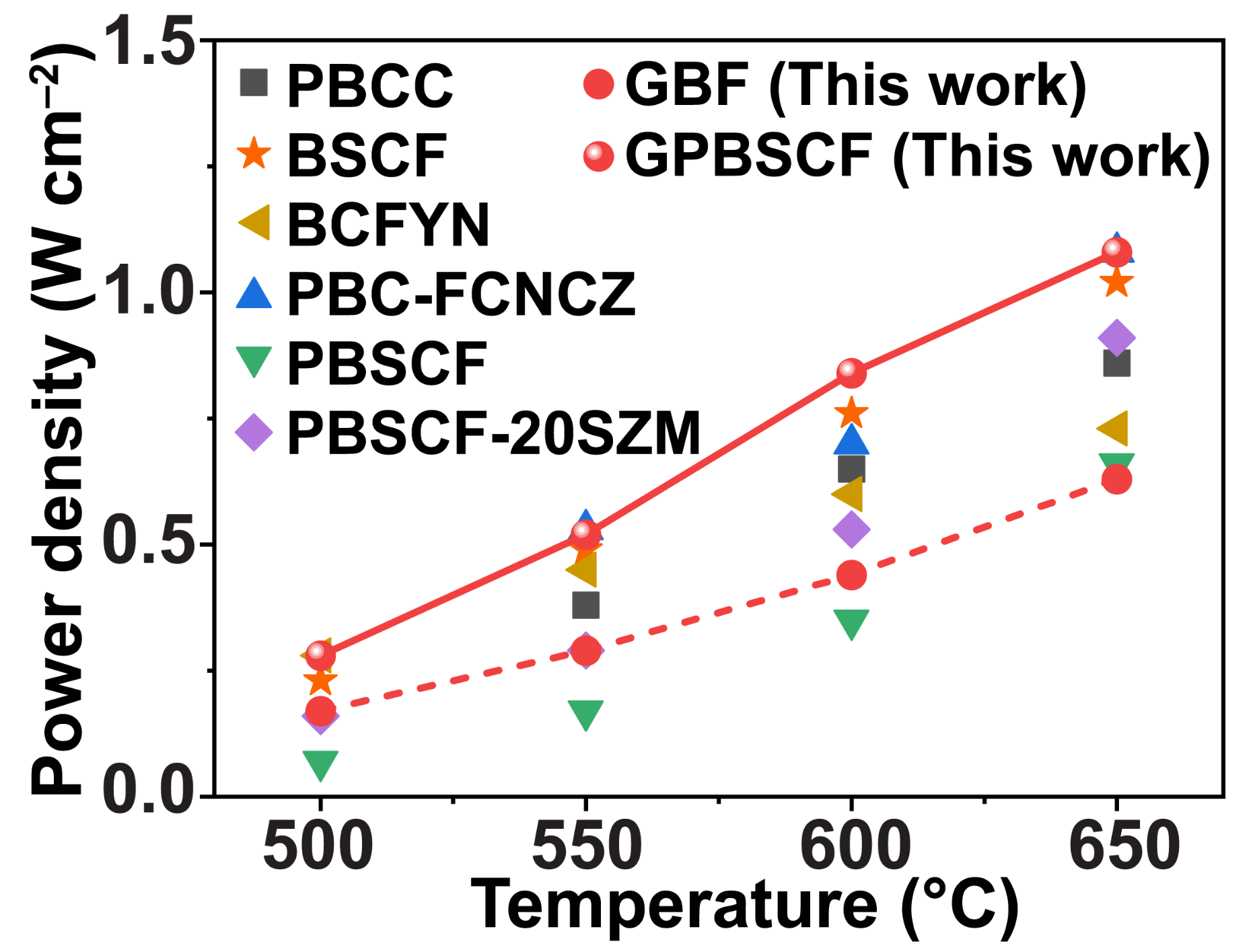


**Figure S20.** PPD comparison of GPBSCF with other air electrode materials in SDC-based single cell.


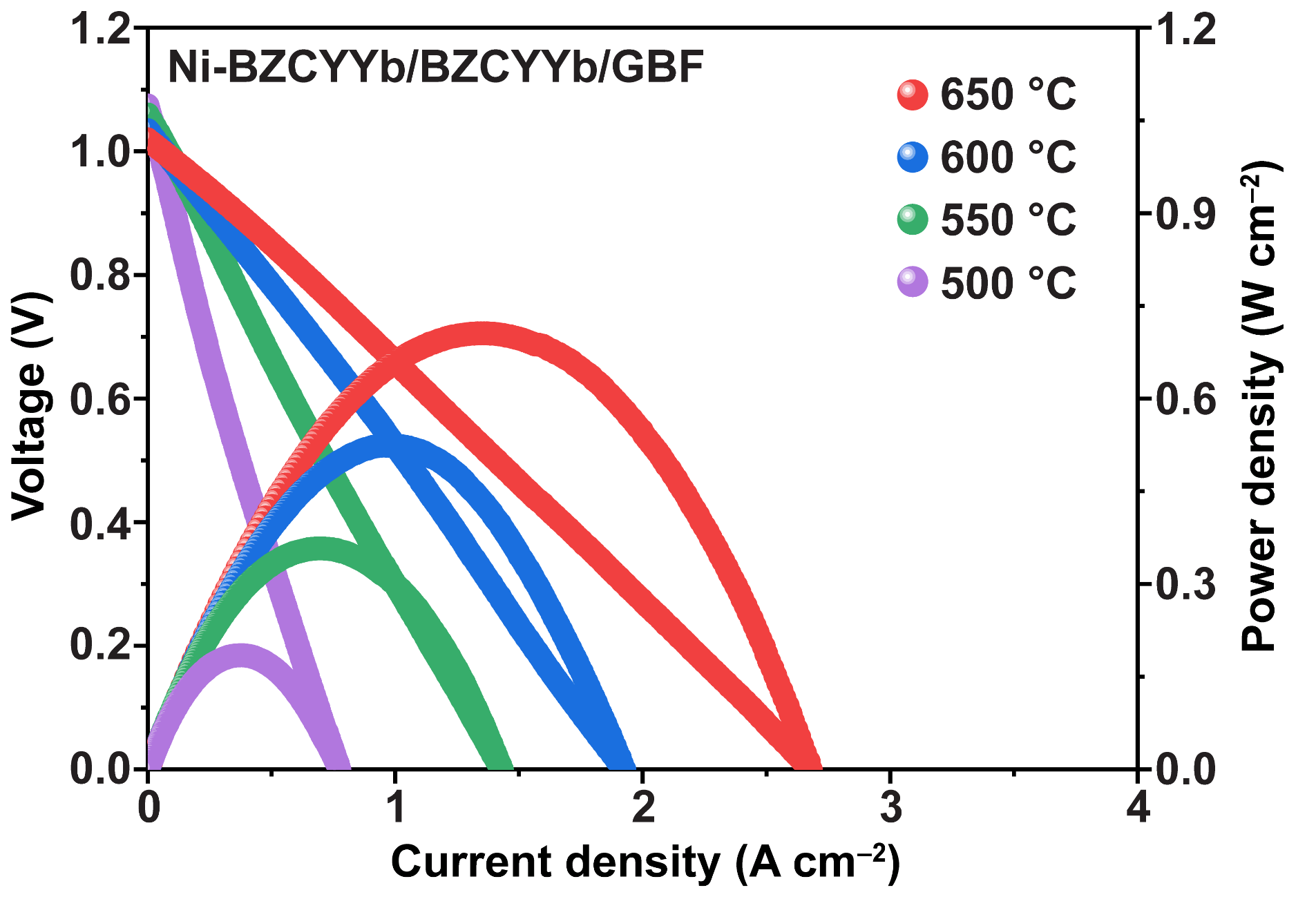


**Figure S21.** Typical *I-V-P* curves of the BZCYYb-based single cell with GBF air electrode measured at 500−650 °C.


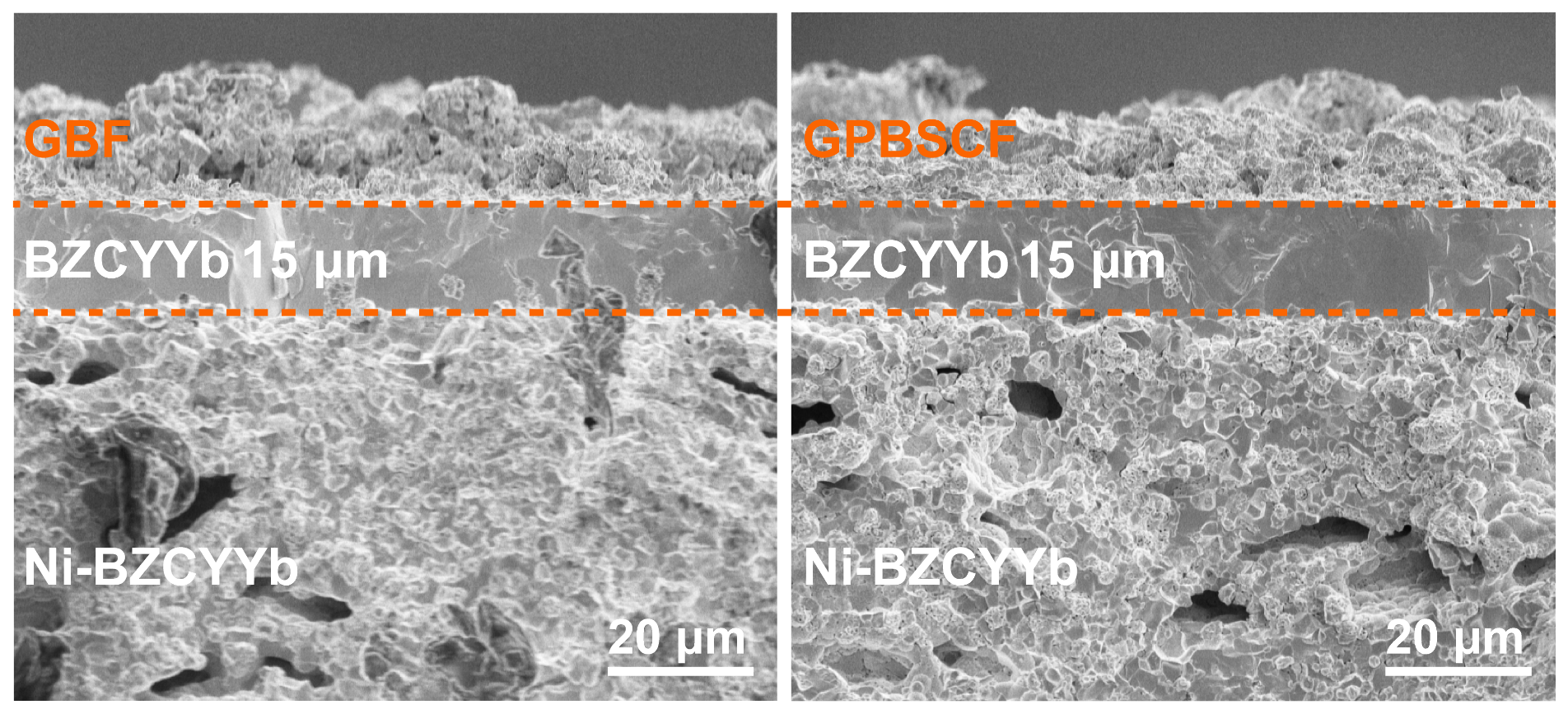


**Figure S22.** Typical cross-sectional SEM images of the Ni-BZCYYb electrode-supported single cells with GBF and GPBSCF air electrodes.


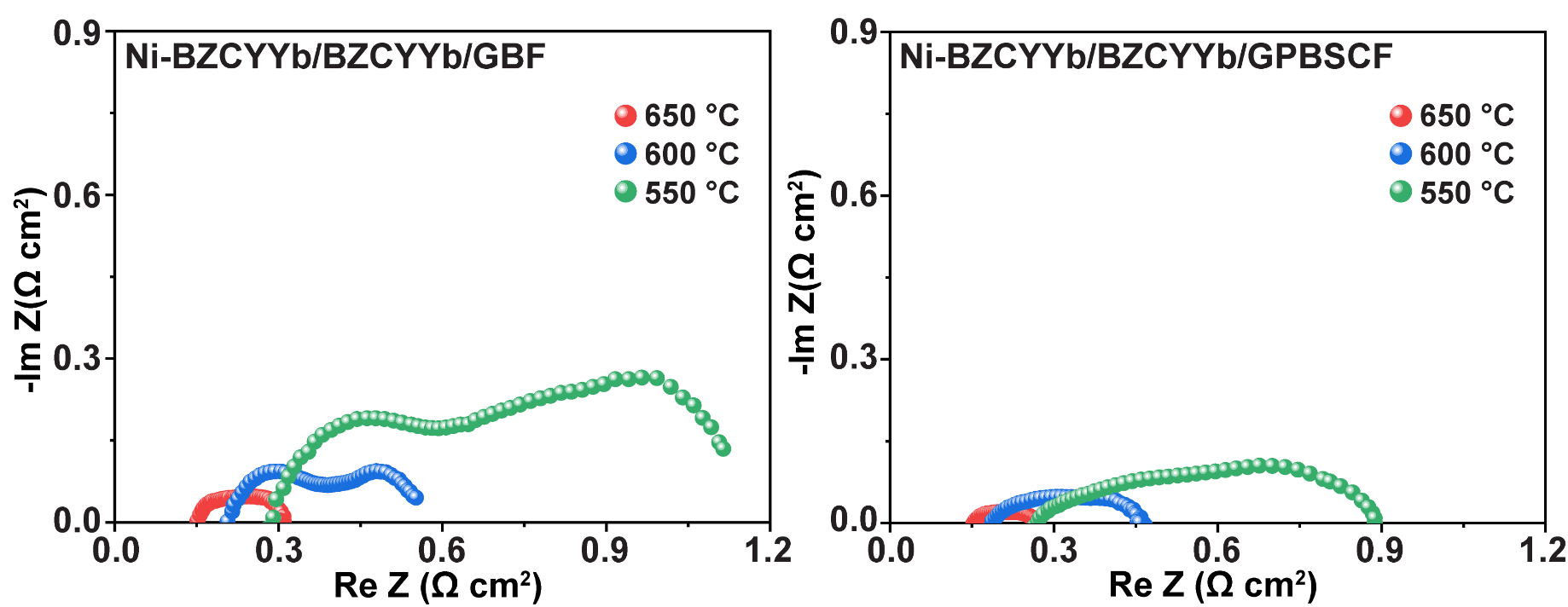


**Figure S23.** Electrochemical impedance spectra (EIS) of the BZCYYb-based single cells with GBF and GPBSCF air electrodes measured at 550−650 °C.


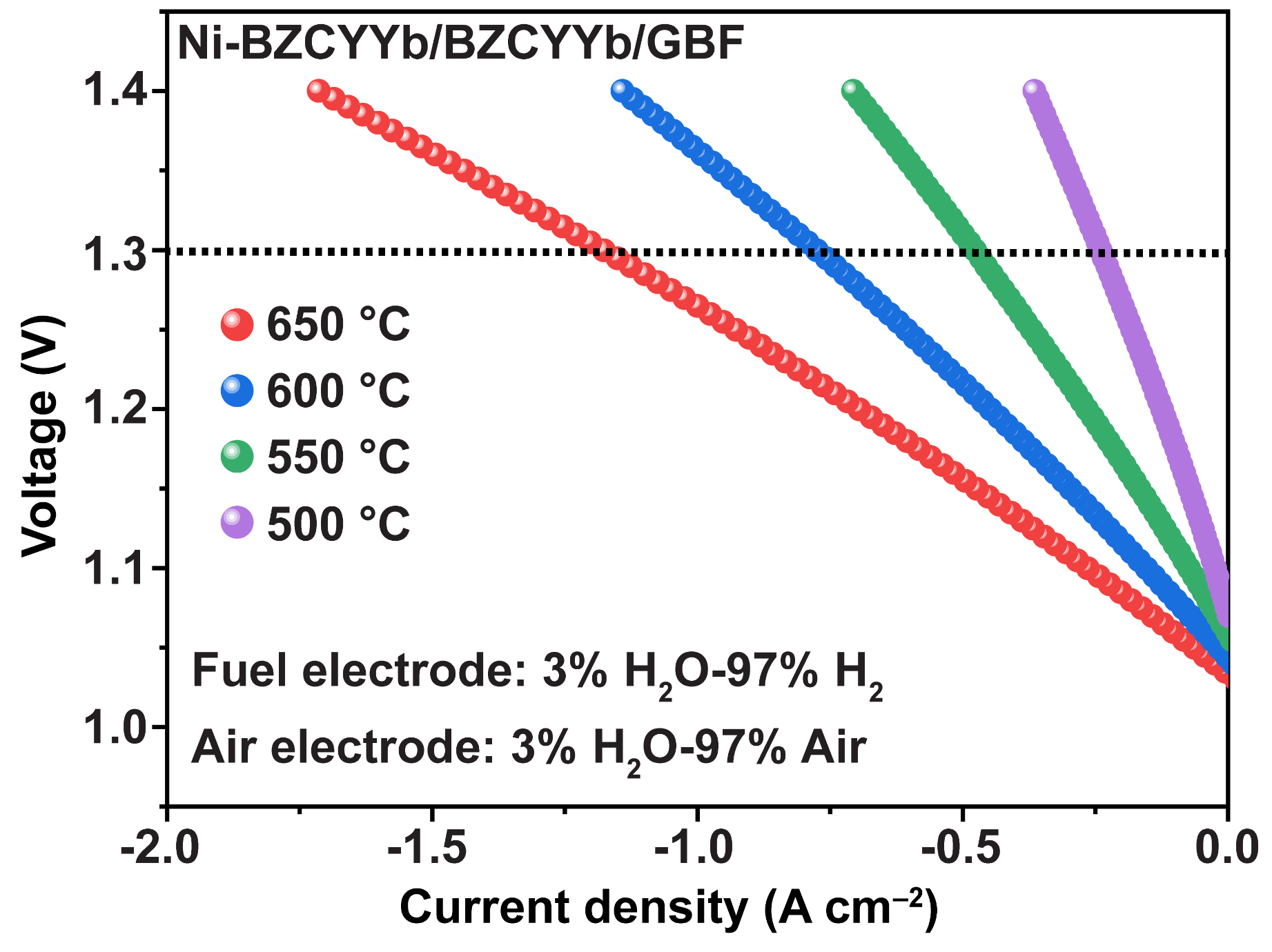


**Figure S24.** *I-V* curves of the BZCYYb-based single cell with GBF air electrode measured at 500−650 °C.


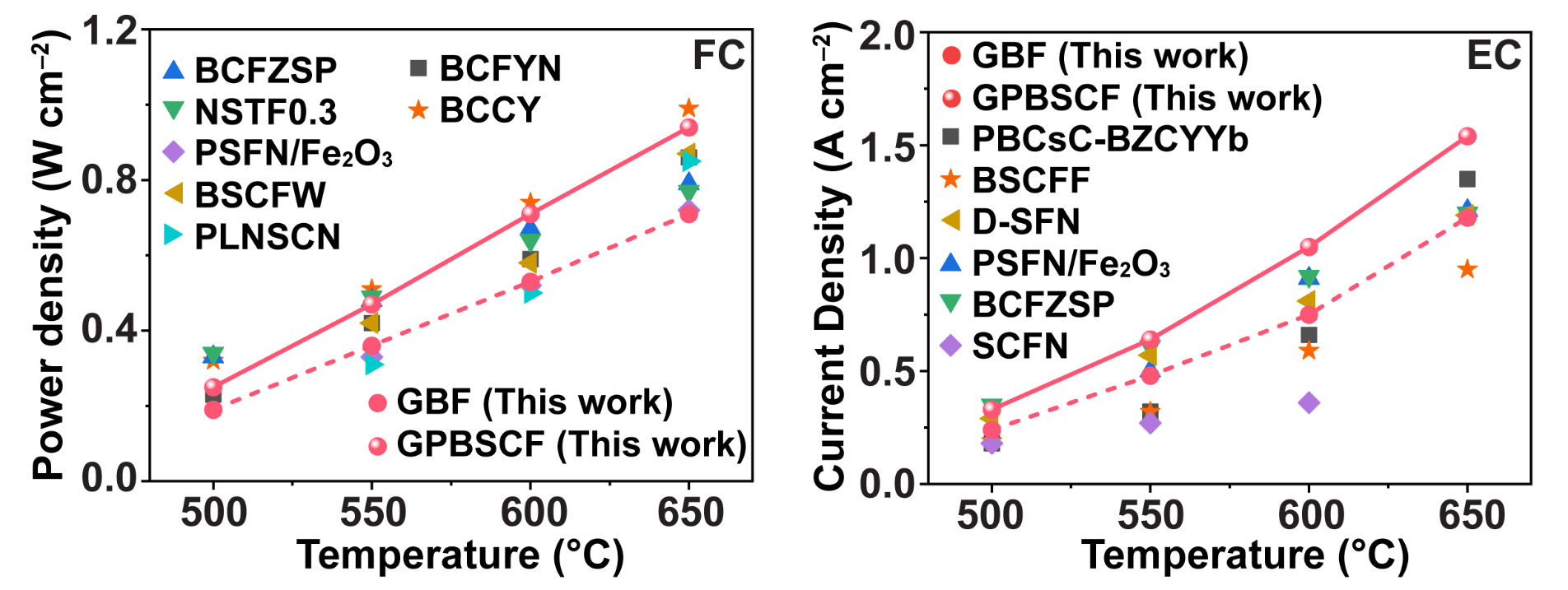


**Figure S25.** PPD and current densities comparison of GPBSCF with other air electrode materials in BZCYYb-based single cell.

**
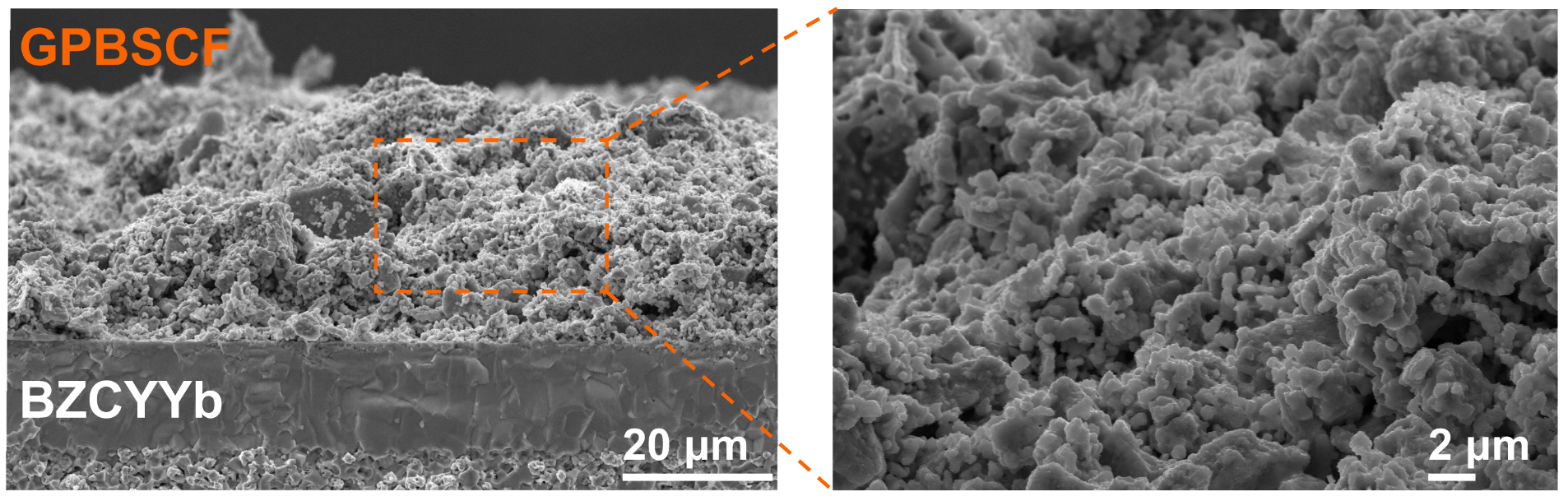
**

**Figure S26.** Morphology of the BZCYYb-based single cell and GPBSCF air electrode after reversible stability test.

**Table S1.** Results of XRD refinements.

| Sample | Space group | a (Å) | b (Å) | c (Å) | Element | Wyck. | Occup | Uiso |
| --- | --- | --- | --- | --- | --- | --- | --- | --- |
| GBF | P4/mmm | 3.937 | 3.937 | 7.748 | Gd | 1b | 0.9879 | 0.02082 |
|  |  |  |  |  | Ba | 1a | 1.0118 18 | 0.02082 |
|  |  |  |  |  | Fe1 | 2h | 0.8015 | 0.01184 |
|  |  |  |  |  | Fe2 | 2h | 0.1985 | 0.01184 |
|  |  |  |  |  | O1 | 1c | 1.0169 | 0.01042 |
|  |  |  |  |  | O2 | 4i | 1.0000 | 0.01042 |
|  |  |  |  |  | O3 | 1d | 0.5056 | 0.01042 |
| GPBSCF | Pm-3m | 3.905 | 3.905 | 3.905 | Gd | 1b | 0.2000 | 0.03633 |
|  |  |  |  |  | Pr | 1b | 0.2000 | 0.03633 |
|  |  |  |  |  | Ba | 1b | 0.2000 | 0.03633 |
|  |  |  |  |  | Sr | 1b | 0.2000 | 0.03633 |
|  |  |  |  |  | Ca | 1b | 0.2000 | 0.03633 |
|  |  |  |  |  | Fe1 | 1a | 0.7522 | 0.02945 |
|  |  |  |  |  | Fe2 | 1a | 0.2478 | 0.02945 |
|  |  |  |  |  | O | 3c | 0.9767 | 0.01613 |

**Table S2.** Relative proportion of the Fe^3+^ and Fe^4+^ obtained by fitting Fe 2p spectra of GBF and GPBSCF samples.

| Valence State | GBF [%] | GPBSCF [%] |
| --- | --- | --- |
| Fe^3+^ | 79.07 | 76.87 |
| Fe^4+^ | 20.93 | 23.13 |

**Table S3.** Relative proportion of the O_lat_, O_ads_ and H_2_O by fitting O 1s spectra of GBF and GPBSCF samples.

| Oxygen Species | GBF [%] | GPBSCF [%] |
| --- | --- | --- |
| O_lat_ | 34.23 | 27.53 |
| O_ads_ | 58.81 | 63.94 |
| H_2_O | 6.96 | 8.53 |

**Table S4.** *K*_chem_ and *D*_chem_ for GBF and GPBSCF samples at various temperatures.

| Temp. | GBF | | GPBSCF | |
| --- | --- | --- | --- | --- |
|  | *K*_chem_  (cm s^−1^) | *D*_chem_  (cm^2^ s^−1^) | *K*_chem_  (cm s^−1^) | *D*_chem_  (cm^2^ s^−1^) |
| 600 °C | 4.18×10^−3^ | 4.22×10^−4^ | 7.44×10^−3^ | 7.14×10^−4^ |
| 550 °C | 1.95×10^−3^ | 1.91×10^−4^ | 5.31×10^−3^ | 4.92×10^−4^ |
| 500 °C | 1.03×10^−3^ | 9.88×10^−5^ | 3.55×10^−3^ | 3.41×10^−4^ |

**Table S5.** Comparison of ASR values of high-performance air electrodes based on SDC electrolyte supported symmetrical cells in dry air.

| Air Electrode | ASR (Ω cm^2^) | | | | Reference |
| --- | --- | --- | --- | --- | --- |
|  | Temperature (^o^C) | | | |  |
|  | 650 | 600 | 550 | 500 |  |
| SFM_0.07_ | 0.73 | 1.65 |  |  | 6 |
| PBSC | 0.23 | 0.60 | 1.38 | 5.70 | 7 |
| PBC-FCNCZ | 0.12 | 0.27 | 0.76 |  | 8 |
| SFNT | 0.15 | 0.28 | 0.63 |  | 9 |
| BCO-LSCF | 0.09 | 0.20 | 0.57 | 1.66 | 10 |
| LSCF | 0.21 | 0.67 | 2.03 | 8.29 | 10 |
| GBF | 0.25 | 0.57 | 1.73 | 6.33 | This work |
| GPBSCF | 0.08 | 0.17 | 0.39 | 1.21 | This work |

Notes: SFM_0.07_: SrFe_0.93_Mo_0.07_O_3–δ_; PBSC: PrBa_0.5_Sr_0.5_Co_2_O_5+δ_; SFNT: SrFe_0.8_Nb_0.1_Ta_0.1_O_3−δ_; BCO-LSCF: barium cobaltite (BaCoO_3-δ_) coated LSCF; PBC-FCNCZ: PrBa_0.8_Ca_0.2_Fe_0.4_Co_0.4_Ni_0.4_Cu_0.4_Zn_0.4_O_6−δ_.

**Table S6.** Comparison of ASR values of high-performance air electrodes based on BZCYYb electrolyte supported symmetrical cells in wet air.

| Air Electrode | ASR (Ω cm^2^) | | | | Reference |
| --- | --- | --- | --- | --- | --- |
|  | Temperature (^o^C) | | | |  |
|  | 650 | 600 | 550 | 500 |  |
| BCFZY | 0.35 | 0.62 | 1.05 | 1.83 | 11 |
| 2W-PBSCF | 0.24 | 0.36 | 0.80 | 1.39 | 12 |
| PFC-PBSCF | 0.25 | 0.81 | 1.88 |  | 13 |
| BCYF | 0.60 | 1.02 | 1.88 | 3.85 | 14 |
| NBSCFN5 | 0.71 | 1.58 | 4.26 | 11.33 | 15 |
| BZFN-BZCY | 0.81 | 1.98 | 5.66 |  | 16 |
| GBF | 0.26 | 0.55 | 1.27 | 3.73 | This work |
| GPBSCF | 0.19 | 0.44 | 0.99 | 2.66 | This work |

Notes: PFC-PBSCF: Pr_0.9_Fe_0.7_Co_0.3_O_3_ surface coating on PBSCF; BCYF: BaCe_0.16_Y_0.04_Fe_0.8_O_3−δ_; NBSCFN5: NdBa_0.5_Sr_0.5_Co_1.5_Fe_0.45_Ni_0.05_O_5+δ_; 2W-PBSCF: (NH_4_)_10_W_12_O_41_·5H_2_O: PBSCF; BCFZY: BaCo_0.4_Fe_0.4_Zr_0.1_Y_0.1_O_3−δ_; BZFN-BZCY: BaZr_0.1_Fe_0.9-x_Ni_x_O_3−δ_-BaZr_0.1_Ce_0.7_Y_0.2_O_3−δ_.

**Table S7.** Comparison of PPD values of high-performance air electrodes based on SDC electrolyte single cells in dry air.

| Air Electrode | Electrolyte | PPD (W cm^–2^) | | | | Reference |
| --- | --- | --- | --- | --- | --- | --- |
|  |  | Temperature (^o^C) | | | |  |
|  |  | 650 | 600 | 550 | 500 |  |
| PBCC | YSZ/GDC | 0.86 | 0.65 | 0.38 |  | 8 |
| PBC-FCNCZ | YSZ/GDC | 1.08 | 0.70 | 0.53 |  | 8 |
| BSCF | SDC | 1.02 | 0.76 | 0.49 | 0.23 | 17 |
| PBSCF | YSZ/GDC | 0.66 | 0.35 | 0.17 | 0.07 | 18 |
| PBSCF-20SZM | YSZ/GDC | 0.91 | 0.53 | 0.29 | 0.16 | 18 |
| BCFYN | SDC | 0.73 | ~ 0.60 | ~ 0.45 | ~ 0.28 | 19 |
| GBF | SDC | 0.63 | 0.44 | 0.29 | 0.17 | This work |
| GPBSCF | SDC | 1.08 | 0.84 | 0.52 | 0.28 | This work |

Notes: PBCC: PrBa_0.8_Ca_0.2_Co_2_O_6−δ_; BSCF: Ba_0.5_Sr_0.5_Co_0.8_Fe_0.2_O_3−δ_; PBSCF: PrBa_0.5_Sr_0.5_Co_1.5_Fe_0.5_O_5+δ_; BCFYN: BaCo_0.6_Fe_0.2_Y_0.1_Nb_0.1_O_3-δ_; PBSCF-20SZM: 80%PrBa_0.5_Sr_0.5_Co_1.5_Fe_0.5_O_5+δ_-20%Sm_0.85_Zn_0.15_MnO_3_; PBC-FCNCZ: PrBa_0.8_Ca_0.2_Fe_0.4_Co_0.4_Ni_0.4_Cu_0.4_Zn_0.4_O_6−δ_.

**Table S8.** Comparison of PPD values of high-performance air electrodes based on BZCYYb electrolyte single cells in dry air.

| Air Electrode | Electrolyte | PPD (W cm^–2^) | | | | Reference |
| --- | --- | --- | --- | --- | --- | --- |
|  |  | Temperature (^o^C) | | | |  |
|  |  | 650 | 600 | 550 | 500 |  |
| BCFYN | BZCYYb | 0.86 | ~ 0.59 | ~ 0.42 | ~ 0.23 | 19 |
| BCCY | BZCYYb | 0.99 | 0.74 | 0.51 | 0.32 | 20 |
| BCFZSP | BZCYYb | 0.79 | 0.67 | 0.48 | 0.33 | 21 |
| NSTF0.3 | BZCYYb | 0.77 | 0.64 | 0.49 | 0.34 | 22 |
| PSFN/Fe_2_O_3_ | BZCYYb | 0.72 | 0.52 | 0.33 |  | 23 |
| BSCFW | BZCYYb | 0.87 | 0.58 | 0.42 |  | 24 |
| PLNSCN | BZCYYb | 0.85 | 0.5 | 0.31 |  | 25 |
| GBF | BZCYYb | 0.71 | 0.53 | 0.36 | 0.19 | This work |
| GPBSCF | BZCYYb | 0.94 | 0.71 | 0.47 | 0.25 | This work |

Notes: BCFYN: BaCo_0.6_Fe_0.2_Y_0.1_Nb_0.1_O_3−δ_; BCCY: BaCo_0.7_(Ce_0.8_Y_0.2_)_0.3_O_3−δ_; BCFZSP: BaCo_0.2_Fe_0.2_Zr_0.2_Sn_0.2_Pr_0.2_O_3−δ_; NSTF0.3: Na_0.3_Sr_0.7_Ti_0.1_Fe_0.9_O_3−δ_; BSCFW: Ba_0.5_Sr_0.5_(Co_0.7_Fe_0.3_)_0.6875_W_0.3125_O_3−δ_; PLNSCN: (Pr_0.2_La_0.2_Nd_0.2_Sr_0.2_Ca_0.2_)_2_NiO_4+δ_; PSFN/Fe_2_O_3_: Pr_0.4_Sr_0.6_Fe_0.9_Nb_0.1_O_3-δ_/α-Fe_2_O_3−δ_.

**Table S9.** Comparison of the current densities at 1.3V of the cell with GPBSCF as air electrode with other recently reported cells.

| Air Electrode | Electrolyte | Current Density@ 1.3 V (A cm^−2^) | | | | Reference |
| --- | --- | --- | --- | --- | --- | --- |
|  |  | Temperature (^o^C) | | | |  |
|  |  | 650 | 600 | 550 | 500 |  |
| PBCsC-BZCYYb | BZCYYb | 1.35 | 0.66 | 0.32 | 0.18 | 26 |
| PSFN/Fe_2_O_3_ | BZCYYb | 1.21 | 0.91 | ~ 0.50 |  | 23 |
| BCFZSP | BZCYYb | 1.20 | 0.92 | 0.61 | 0.35 | 21 |
| SCFN | BZCYYb |  | 0.36 | 0.27 | 0.18 | 27 |
| D-SFN | BZCYYb | 1.19 | 0.81 | 0.57 | 0.29 | 28 |
| BSCFF | BZCYYb | 0.95 | 0.59 | 0.32 | 0.19 | 29 |
| GBF | BZCYYb | 1.18 | 0.75 | 0.48 | 0.24 | This work |
| GPBSCF | BZCYYb | 1.54 | 1.05 | 0.64 | 0.33 | This work |

Notes: BSCFF: Ba_0.5_Sr_0.5_Co_0.8_Fe_0.2_O_2.9-δ_F_0.1_; SCFN: Sr_0.9_Ce_0.1_Fe_0.8_Ni_0.2_O_3-δ_; BCFZSP: BaCo_0.2_Fe_0.2_Zr_0.2_Sn_0.2_Pr_0.2_O_3-δ_; PSFN/Fe_2_O_3_: Pr_0.4_Sr_0.6_Fe_0.9_Nb_0.1_O_3-δ_/α-Fe_2_O_3-δ_; D-SFN: Sr_2.8_Fe_1.8_Nb_0.2_O_7−δ_; PBCsC: PrBa_0.875_Cs_0.125_Co_2_O_5+δ_.

**References**

1. G. Kresse, J. Furthmüller, Efficiency of ab-initio total energy calculations for metals and semiconductors using a plane-wave basis set, *Computational Materials Science* 6(1) (1996) 15-50. https://doi.org/10.1016/0927-0256(96)00008-0.

2. G. Kresse, J. Furthmüller, Efficient iterative schemes for ab initio total-energy calculations using a plane-wave basis set, *Physical Review B* 54(16) (1996) 11169-11186. https://doi.org/10.1103/PhysRevB.54.11169.

3. J.P. Perdew, K. Burke, M. Ernzerhof, Generalized Gradient Approximation Made Simple, *Physical Review Letters* 77(18) (1996) 3865-3868. https://doi.org/10.1103/PhysRevLett.77.3865.

4. P.E. Blöchl, Projector augmented-wave method, *Physical Review B* 50(24) (1994) 17953-17979. https://doi.org/10.1103/PhysRevB.50.17953.

5. X. Xu, H. Wang, M. Fronzi, X. Wang, L. Bi, E. Traversa, Tailoring cations in a perovskite cathode for proton-conducting solid oxide fuel cells with high performance, *Journal of Materials Chemistry A* 7(36) (2019) 20624-20632. https://doi.org/10.1039/C9TA05300J.

6. Y. Li, N. Mushtaq, Y. Chen, W. Ye, Z. Zhuang, M. Singh, Y. Jing, L. Fan, Revisiting Mo-Doped SrFeO_3-δ_ Perovskite: The Origination of Cathodic Activity and Longevity for Intermediate-Temperature Solid Oxide Fuel Cells, *Advanced Functional Materials* 35(3) (2025) 2411025. https://doi.org/10.1002/adfm.202411025.

7. K. Liu, F. Lu, X. Jia, H. He, J. Su, B. Cai, A high performance thermal expansion offset composite cathode for IT-SOFCs, *Journal of Materials Chemistry A* 10(45) (2022) 24410-24421. https://doi.org/10.1039/D2TA04899J.

8. Z. Xia, Y. Zhang, X. Xiong, J. Cui, Z. Liu, S. Xi, Z. Hu, J.-Q. Wang, L. Zhang, Realizing B-site high-entropy air electrode for superior reversible solid oxide cells, *Applied Catalysis B: Environment and Energy* 357 (2024) 124314. https://doi.org/10.1016/j.apcatb.2024.124314.

9. B. Admasu Beshiwork, X. Wan, M. Xu, H. Guo, B. Sirak Teketel, Y. Chen, J. Song Chen, T. Li, E. Traversa, A defective iron-based perovskite cathode for high-performance IT-SOFCs: Tailoring the oxygen vacancies using Nb/Ta co-doping, *Journal of Energy Chemistry* 88 (2024) 306-316. https://doi.org/10.1016/j.jechem.2023.09.015.

10. K. Pei, Y. Zhou, K. Xu, Z. He, Y. Chen, W. Zhang, S. Yoo, B. Zhao, W. Yuan, M. Liu, Y. Chen, Enhanced Cr-tolerance of an SOFC cathode by an efficient electro-catalyst coating, *Nano Energy* 72 (2020) 104704. https://doi.org/10.1016/j.nanoen.2020.104704.

11. C. Duan, J. Tong, M. Shang, S. Nikodemski, M. Sanders, S. Ricote, A. Almansoori, R. O’Hayre, Readily processed protonic ceramic fuel cells with high performance at low temperatures, *Science* 349(6254) (2015) 1321-1326. https://doi.org/10.1126/science.aab3987.

12. S. Zhao, W. Ma, W. Wang, Y. Huang, J. Wang, S. Wang, Z. Shu, B. He, L. Zhao, Reverse Atom Capture on Perovskite Surface Enabling Robust and Efficient Cathode for Protonic Ceramic Fuel Cells, *Advanced Materials* 36(27) (2024) 2405052. https://doi.org/10.1002/adma.202405052.

13. H. Zhang, K. Xu, F. He, Y. Zhou, K. Sasaki, B. Zhao, Y. Choi, M. Liu, Y. Chen, Surface Regulating of a Double-Perovskite Electrode for Protonic Ceramic Fuel Cells to Enhance Oxygen Reduction Activity and Contaminants Poisoning Tolerance, *Advanced Energy Materials* 12(26) (2022) 2200761. https://doi.org/10.1002/aenm.202200761.

14. D. Zou, Y. Yi, Y. Song, D. Guan, M. Xu, R. Ran, W. Wang, W. Zhou, Z. Shao, The BaCe_0.16_Y_0.04_Fe_0.8_O_3−δ_ nanocomposite: a new high-performance cobalt-free triple-conducting cathode for protonic ceramic fuel cells operating at reduced temperatures, *Journal of Materials Chemistry A* 10(10) (2022) 5381-5390. https://doi.org/10.1039/D1TA10652J.

15. G.-M. Park, K. Park, M. Jo, M. Asif, Y. Bae, S.-H. Kim, A.K. Azad, S.-J. Song, J.-Y. Park, Nickel-doped NdBa_0.5_Sr_0.5_Co_1.5_Fe_0.5_O_5+δ_ oxygen electrode material for high performance reversible protonic ceramic cells, *Journal of Alloys and Compounds* 968 (2023) 171987. https://doi.org/10.1016/j.jallcom.2023.171987.

16. J. Wang, Z. Li, H. Zang, Y. Sun, Y. Zhao, Z. Wang, Z. Zhu, Z. Wei, Q. Zheng, BaZr_0.1_Fe_0.9-x_Ni_x_O_3-δ_ cubic perovskite oxides for protonic ceramic fuel cell cathodes, *International Journal of Hydrogen Energy* 47(15) (2022) 9395-9407. https://doi.org/10.1016/j.ijhydene.2022.01.012.

17. Y. Zhang, Y. Wang, Z. Liu, Z. Wang, Y. Wang, Y. Xiao, B. Niu, X. Wang, W. Wang, T. He, Constructing Robust and Efficient Ceramic Cells Air Electrodes Through Collaborative Optimization Bulk and Surface Phases, *Advanced Functional Materials* 35(20) (2025) 2422531. https://doi.org/10.1002/adfm.202422531.

18. X. Liu, X. Xi, Y. Liao, L. Huang, J. Liu, H. Chen, Y. Yi, J. Long, J. Zhang, X.-Z. Fu, J.-L. Luo, Deciphering the enhanced oxygen reduction reaction activity of PrBa_0.5_Sr_0.5_Co_1.5_Fe_0.5_O_5+δ_ via constructing negative thermal expansion offset for high-performance solid oxide fuel cell, *Applied Catalysis B: Environment and Energy* 359 (2024) 124509. https://doi.org/10.1016/j.apcatb.2024.124509.

19. Z. Liu, H. Xie, Y. Zhang, J. Li, J. You, H. Yang, H. Zhu, M. Ni, Z. Shao, B. Chen, Towards high performance durable ceramic fuel cells using a triple conducting perovskite cathode, *Applied Catalysis B: Environment and Energy* 346 (2024) 123678. https://doi.org/10.1016/j.apcatb.2023.123678.

20. Y. Song, Y. Chen, W. Wang, C. Zhou, Y. Zhong, G. Yang, W. Zhou, M. Liu, Z. Shao, Self-Assembled Triple-Conducting Nanocomposite as a Superior Protonic Ceramic Fuel Cell Cathode, *Joule* 3(11) (2019) 2842-2853. https://doi.org/10.1016/j.joule.2019.07.004.

21. J. Sun, R. Ren, H. Yue, W. Cui, G. Wang, C. Xu, J. Qiao, W. Sun, K. Sun, Z. Wang, High-entropy perovskite oxide BaCo_0.2_Fe_0.2_Zr_0.2_Sn_0.2_Pr_0.2_O_3-δ_ with triple conduction for the air electrode of reversible protonic ceramic cells, *Chinese Chemical Letters* 34(7) (2023) 107776. https://doi.org/10.1016/j.cclet.2022.107776.

22. C. Zhou, X. Wang, D. Liu, M. Fei, J. Dai, D. Guan, Z. Hu, L. Zhang, Y. Wang, W. Wang, R. O'Hayre, S.P. Jiang, W. Zhou, M. Liu, Z. Shao, New Strategy for Boosting Cathodic Performance of Protonic Ceramic Fuel Cells Through Incorporating a Superior Hydronation Second Phase, *Energy & Environmental Materials* 7(4) (2024) e12660. https://doi.org/10.1002/eem2.12660.

23. Z. Wang, Y. Xiao, Y. Zhang, Y. Wang, X. Wang, F. Wang, T. He, A novel chemical composite strategy for synergistically enhancing the activity and stability of strontium-containing air electrode for protonic ceramic cells, *Chemical Engineering Journal* 490 (2024) 151911. https://doi.org/10.1016/j.cej.2024.151911.

24. D. Hu, J. Kim, H. Niu, L.M. Daniels, T.D. Manning, R. Chen, B. Liu, R. Feetham, J.B. Claridge, M.J. Rosseinsky, High-performance protonic ceramic fuel cell cathode using protophilic mixed ion and electron conducting material, *Journal of Materials Chemistry A* 10(5) (2022) 2559-2566. https://doi.org/10.1039/D1TA07113K.

25. K. Kang, Y. Liu, X. Liu, C. Wang, M. Wei, Local lattice distortion regulation in high entropy engineering to enhance the triple conductivity of layered Ruddlesden-Popper perovskite cathode in H^+^-SOFCs, *Chemical Engineering Journal* 507 (2025) 159463. https://doi.org/10.1016/j.cej.2025.159463.

26. N. Shi, K. Zhu, Y. Xie, D. Huan, J. Hyodo, Y. Yamazaki, Investigation of Water Impacts on Surface Properties and Performance of Air-Electrode in Reversible Protonic Ceramic Cells, *Small* 20(36) (2024) 2400501. https://doi.org/10.1002/smll.202400501.

27. Y. Song, J. Liu, Y. Wang, D. Guan, A. Seong, M. Liang, M.J. Robson, X. Xiong, Z. Zhang, G. Kim, Z. Shao, F. Ciucci, Nanocomposites: A New Opportunity for Developing Highly Active and Durable Bifunctional Air Electrodes for Reversible Protonic Ceramic Cells, *Advanced Energy Materials* 11(36) (2021) 2101899. https://doi.org/10.1002/aenm.202101899.

28. N. Yu, I.T. Bello, X. Chen, T. Liu, Z. Li, Y. Song, M. Ni, Rational Design of Ruddlesden–Popper Perovskite Ferrites as Air Electrode for Highly Active and Durable Reversible Protonic Ceramic Cells, *Nano-Micro Letters* 16(1) (2024) 177. https://doi.org/10.1007/s40820-024-01397-2.

29. X. Chen, N. Yu, I.T. Bello, D. Guan, Z. Li, T. Liu, T. Liu, Z. Shao, M. Ni, Facile anion engineering: A pathway to realizing enhanced triple conductivity in oxygen electrodes for reversible protonic ceramic electrochemical cells, *Energy Storage Materials* 63 (2023) 103056. https://doi.org/10.1016/j.ensm.2023.103056.
